# Leveraging deep single-soma RNA sequencing to explore the neural basis of human somatosensation

**DOI:** 10.1038/s41593-024-01794-1

**Published:** 2024-11-04

**Authors:** Huasheng Yu, Saad S. Nagi, Dmitry Usoskin, Yizhou Hu, Jussi Kupari, Otmane Bouchatta, Hanying Yan, Suna Li Cranfill, Mayank Gautam, Yijing Su, You Lu, James Wymer, Max Glanz, Phillip Albrecht, Hongjun Song, Guo-Li Ming, Stephen Prouty, John Seykora, Hao Wu, Minghong Ma, Andrew Marshall, Frank L. Rice, Mingyao Li, Håkan Olausson, Patrik Ernfors, Wenqin Luo

**Affiliations:** 1 Department of Neuroscience, Perelman School of Medicine, University of Pennsylvania, Philadelphia, PA USA; 2 Department of Biomedical and Clinical Sciences, Linköping University, Linköping, Sweden; 3 Department of Medical Biochemistry and Biophysics, Division of Molecular Neurobiology, Karolinska Institute, Stockholm, Sweden; 4 Department of Biostatistics in Biostatistics and Epidemiology, Perelman School of Medicine, University of Pennsylvania, Philadelphia, PA USA; 5 Department of Biochemistry and Molecular Biology, College of Medicine, University of Florida, Gainesville, FL USA; 6 Neuroscience & Pain Research Group, Integrated Tissue Dynamics, LLC, Rensselaer, NY USA; 7 Department of Dermatology, Perelman School of Medicine, University of Pennsylvania, Philadelphia, PA USA; 8 Department of Genetics, Perelman School of Medicine, University of Pennsylvania, Philadelphia, PA USA; 9 Pain Research Institute, Institute of Life Course and Medical Sciences, University of Liverpool, Liverpool, UK

**Keywords:** Sensory processing, Somatic system

## Abstract

The versatility of somatosensation arises from heterogeneous dorsal root ganglion (DRG) neurons. However, soma transcriptomes of individual human (h)DRG neurons—critical information to decipher their functions—are lacking due to technical difficulties. In this study, we isolated somata from individual hDRG neurons and conducted deep RNA sequencing (RNA-seq) to detect, on average, over 9,000 unique genes per neuron, and we identified 16 neuronal types. These results were corroborated and validated by spatial transcriptomics and RNAscope in situ hybridization. Cross-species analyses revealed divergence among potential pain-sensing neurons and the likely existence of human-specific neuronal types. Molecular-profile-informed microneurography recordings revealed temperature-sensing properties across human sensory afferent types. In summary, by employing single-soma deep RNA-seq and spatial transcriptomics, we generated an hDRG neuron atlas, which provides insights into human somatosensory physiology and serves as a foundation for translational work.

## Abstract (web summary)

Dorsal root ganglia (DRGs) contain a plethora of neuron types. The authors show that the existence of human-specific DRG neuronal types and microneurography recordings reveal distinct temperature-sensing properties across human sensory afferent types.

## Main

Most current knowledge about mammalian somatosensory neurons comes from model organisms. However, the success rate of translating treatment strategies from model organisms to humans, such as those for chronic pain, is low^1,2^. Considerable differences exist between rodent and human dorsal root ganglion (hDRG) neurons^3,4^, underscoring the need to elucidate the molecular profiles and cell types of hDRG neurons.

**Fig. 1:**
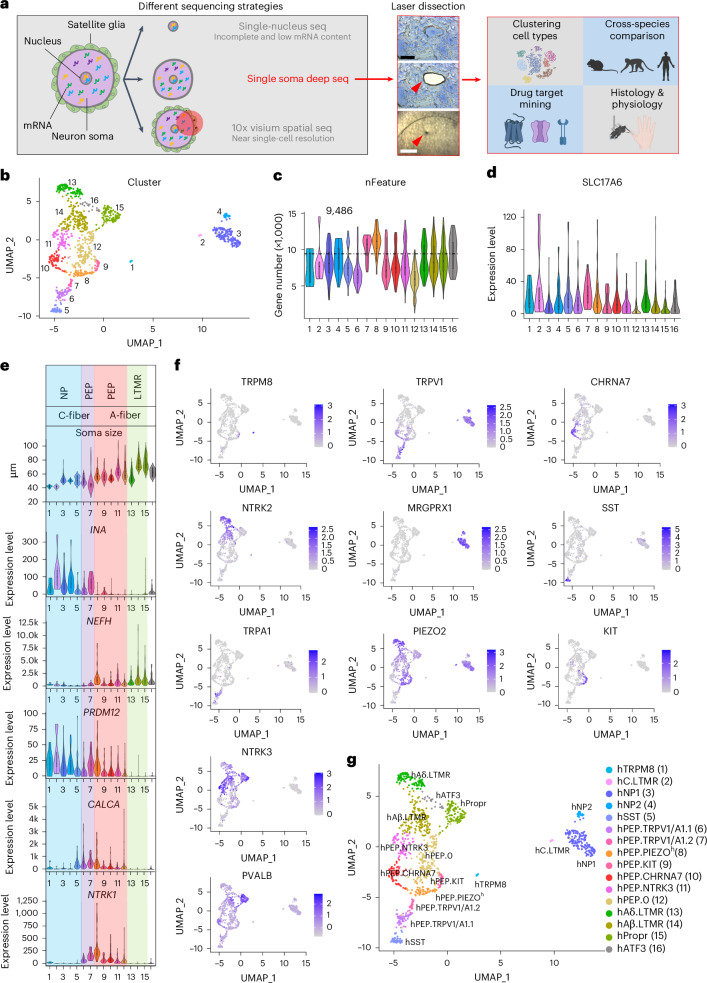
Developing an LCM-based approach for single-soma deep RNA-seq of hDRG neurons. **a**, Overall workflow of this study. Left, features associated with different strategies for single-cell RNA-seq of hDRG neurons. Middle, example of the laser dissection of an hDRG neuron soma. Right, summary of analyses and experiments. Scale bar, 50 μm (cell) and 500 μm (cap). **b**, UMAP plot showing the clusters of 1,066 hDRG neurons. **c**,**d**, Violin plots showing total number of detected genes (**c**) and the expression of neuronal marker *SLC17A6* (**d**). **e**, The grouping clusters based on the top 50% soma diameter and the expression of *INA*, *NEFH*, *PRDM12*, *CALCA* and *NTRK1*. **f**, UMAPs showing some canonical marker gene expression in each cluster. The color gradient represents the log-normalized expression levels of each gene across cells. **g**, UMAP plot with names of each cluster. For violin plots in **c**–**e**, the lower and upper bounds of the box correspond to the first quartile (Q1, 25%) and the third quartile (Q3, 75%), respectively. A gray dot within the box represents the median value (Q2, 50%). The lower end of the whiskers indicates the minimal value within 1.5 times the interquartile range (IQR) from the first quartile (Q1, 25%). The upper end of the whiskers indicates the maximal value within 1.5 times the IQR from the third quartile (Q3, 75%). Source data

Single-cell RNA sequencing (RNA-seq) is a powerful method for studying transcripts of individual cells (soma and/or nuclei) and for classifying heterogenous cells into different types^5,6^. This approach has been effective for studying DRG neurons in mouse and other model organisms^7–10^, but it encounters challenges in humans due to (1) the substantial number of non-neuronal cells in hDRGs^4^ and (2) the large size of hDRG neuronal somata, which makes them prone to damage by enzyme digestion and mechanical forces during single-cell isolation. Enzymatic and mechanical dissociation may also cause transcriptome changes^11^. Owing to these difficulties, single-nucleus RNA-seq and 10x Visium spatial transcriptomics have been employed^12–15^. However, the quantity of nuclear transcripts is lower than that in the soma; the nucleus transcript species may not represent the full transcriptome profile of the cell^16^; and 10x Visium spatial transcriptomics lacks single-neuron resolution (Fig. 1a).

In this study, we developed a strategy combining laser capture microdissection (LCM) for isolating individual neuronal somata with Smart-seq2 (ref. ^17^) for generating full-length RNA-seq libraries (Fig. 1a). We sequenced 1,066 hDRG neurons with minimum satellite glial cell contamination from six lower thoracic and lumbar DRGs of three donors, detecting an average of approximately 9,000 unique genes per neuron and identifying 16 molecularly distinct neuron types. Cross-species analyses revealed similarities as well as considerable differences among human, macaque and mouse DRG neurons. We also uncovered a set of marker genes that distinguished the different sensory neuron types. 10x Xenium spatial transcriptomics confirmed the 16 populations of hDRG neurons and revealed previously unidentified spatial clustering patterns. Based on molecular profiles, we predicted temperature and chemical response properties of various human skin afferents, which were confirmed using human microneurography recordings. Our results reveal a close relationship between the molecular profiles and functional properties of human sensory afferents.

## Results

### An LCM-based method for single-soma RNA-seq of hDRG neurons

**Extended Data Fig. 1:**
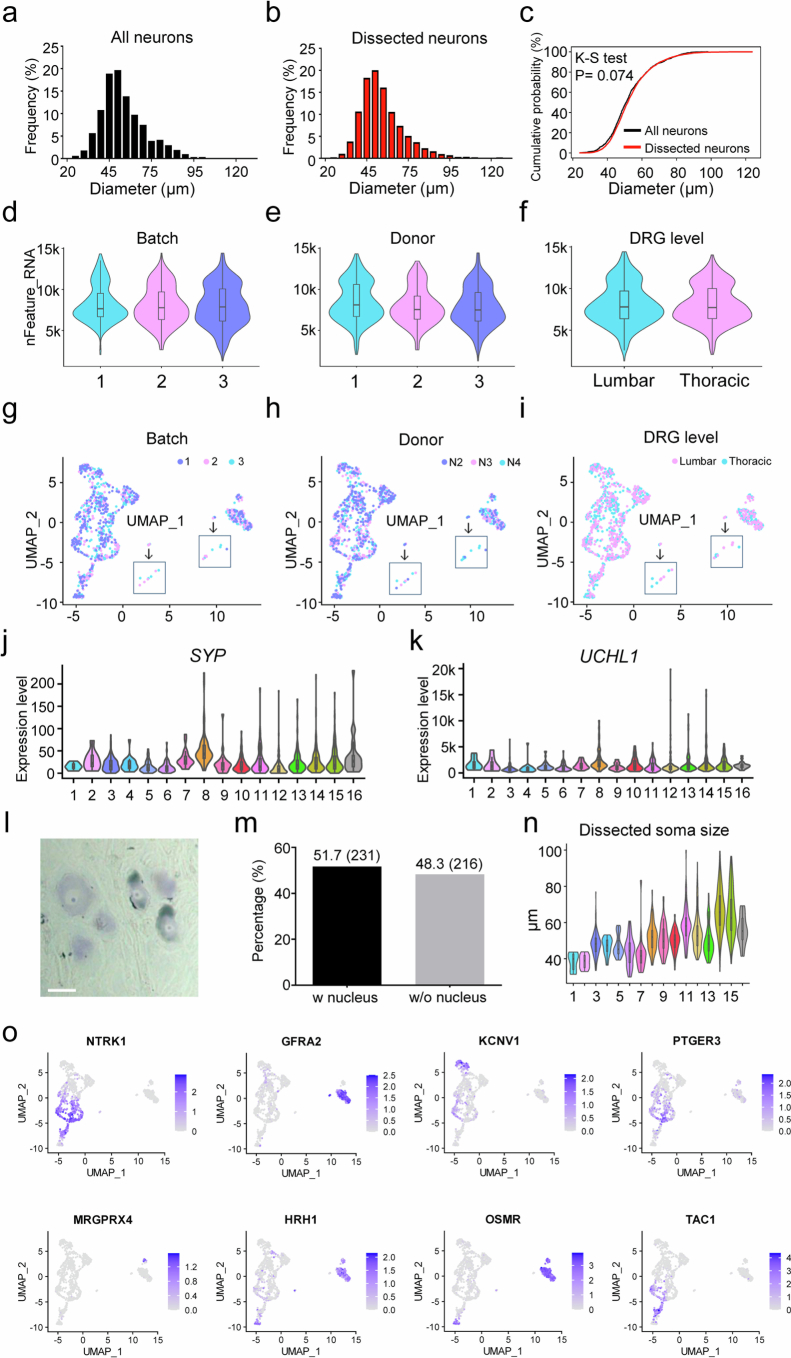
LCM RNA-seq statistics, clustering, and additional marker gene expression. (**a-c**) Size distribution of all DRG neurons (**a**) and dissected neuronal soma (**b**). (**c**) Cumulative curve of size distribution of all DRG neurons in (**a**) and dissected neuronal somata in (**b**) and statistic comparison by two-sided Kolmogorov–Smirnov test. (**d-f**) Violin plots showing total number of detected genes in different batches (**d**), donors (**e**), and DRG levels (**f**). (**g-i**) UMAP plots showing the contribution of individual batches (**g**), donors (**h**) and DRG levels (**i**) to each cluster. The insets in each panel show a zoomed-in view of hTRPM8 and hC.LTMR clusters. (**j-k**) Violin plots showing the expression of pan-neuronal markers *SYP* and *UCHL1*. (**l-m**) A representative image of human DRG neurons stained by HistoGene^TM^ dye (**l**) and quantification of the percentage of neurons with or without the nucleus in 20-μm human DRG tissue sections (**m**, 447 neurons from 10 sections of 5 DRGs, 3 donors). Scale bar, 50 μm. (**n**) The distribution of soma diameters of dissected neurons in each cluster. For violin plots in **d**-**f**, **j**-**k** and **n**, the lower and upper bounds of the box correspond to the first quartile (Q1, 25%) and the third quartile (Q3, 75%), respectively. A grey dot within the box represents the median value (Q2, 50%) The lower end of the whiskers indicates the minimal value within 1.5 times the interquartile range (IQR) from the first quartile (Q1, 25%). The upper end of the whiskers indicates the maximal value within 1.5 times the IQR from the third quartile (Q3, 75%). (**o**) UMAPs showing expression pattern of additional canonical marker genes in each cluster. The color scale represents the log-normalized expression levels of each gene across cells. Source data

Six hDRGs at the low thoracic (T11–T12) and lumbar (L2–L5) levels were extracted from three postmortem donors (Fig. 1a and Supplementary Tables 1 and 2) and immediately frozen in Optimal Cutting Temperature (OCT) compound, cryosectioned, mounted onto LCM slides and briefly stained for cell visualization. Individual neuronal somata were dissected with a laser. Dissected neuronal somata exhibited a diameter distribution similar to the whole DRG neuron population (Extended Data Fig. 1a–c), suggesting that LCM dissections likely sampled all types of DRG neurons. Out of 1,136 sequenced neuronal somata, 70 had glial cell contamination and were removed, so 1,066 neurons were used for analysis. Sixteen transcriptomic clusters of hDRG neurons were identified by Seurat^18^ (Fig. 1b), with an average of 9,486 genes detected per cell (Fig. 1c). No obvious batch effects, donor or DRG level differences were observed in the clusters (Extended Data Fig. 1d–i). All cells expressed peripheral sensory neuronal markers *SLC17A6* (*VGLUT2*), *SYP* (synaptophysin) and *UCHL1* (*PGP9.5*) (Fig. 1d and Extended Data Fig. 1j,k).

### Anatomical and molecular features of hDRG neuron clusters

Mammalian DRG neurons contain myelinated large-diameter (A) and unmyelinated small-diameter (C) afferent fibers^19^, which are further divided into peptidergic and non-peptidergic types based on *CALCA* and *NTRK1* expression^20^. Because about half of the hDRG neurons were sectioned at their middle plane (containing nuclei; Extended Data Fig. 1l,m), the top 50% soma diameters of each cluster were used for cell size estimation and arranged from small to large (Fig. 1e and Extended Data Fig. 1n). Neurofilament intermediate filament protein alpha (*INA*), enriched in small-diameter DRG neurons, and heavy chain (*NEFH*), enriched in large-diameter DRG neurons, showed a complementary pattern. These morphological and molecular features suggested that clusters 1–7 were C-fiber DRG neurons, whereas clusters 8–16 were A-fiber DRG neurons (Fig. 1e). The two groups were further divided based on their co-expression of *CALCA* and *NTRK1* (clusters 6–12). Moreover, *PRDM12*, a transcriptional regulator critical for the development of human C-fibers and pain-sensing afferents (nociceptors)^21^, was expressed in clusters 1–12, further distinguishing them from A-fiber low-threshold mechanoreceptors (A-LTMRs) (Fig. 1e). We deduced that clusters 1–5 were likely non-peptidergic C-fibers, clusters 6–7 were peptidergic C-fibers, clusters 8–12 were peptidergic A-fibers, clusters 13–15 were A-LTMRs and cluster 16 was an uncharacterized A-fiber type (Fig. 1e).

Based on the expression profiles of top molecular markers (Fig. 1f and Extended Data Fig. 1o), we named these 16 hDRG neuron clusters using the following rules (Fig. 1g): (1) ‘human’ was indicated by ‘h’ at the beginning; (2) mouse nomenclature was used for conserved subtypes (that is, A-LTMRs, C-LTMRs, TRPM8 and non-peptidergic (NP) neurons except NP3); and (3) human peptidergic neuron types were designated as hPEP (marker gene). Accordingly, cluster 1 was named hTRPM8; cluster 2 was named hC.LTMR; cluster 3 was named hNP1; cluster 4 was named hNP2; cluster 5 was named hSST; cluster 6 was named hPEP.TRPV1/A1.1; cluster 7 was named hPEP.TRPV1/A1.2; cluster 8 was named hPEP.PIEZO^h^ (the superscript ‘h’ meaning ‘high’); cluster 9 was named hPEP.KIT; cluster 10 was named hPEP.CHRNA7; cluster 11 was named hPEP.NTRK3; cluster 12 was named hPEP.0 (no distinctive molecular marker); cluster 13 was named hAδ.LTMR; cluster 14 was named hAβ.LTMR; cluster 15 was named proprioceptors (hPropr); and cluster 16 was named hATF3.

**Extended Data Fig. 2:**
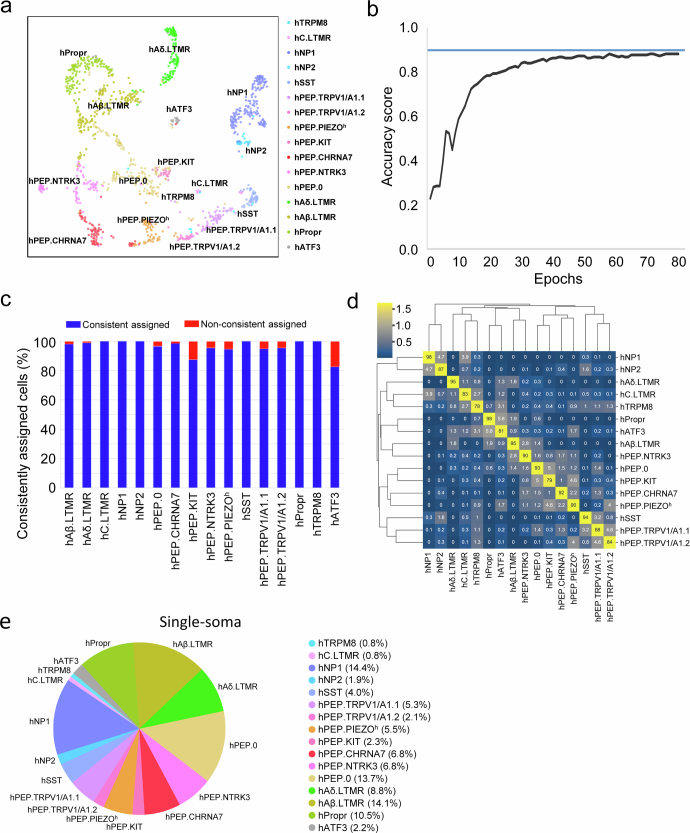
Validation of hDRG neuron clustering. (**a**) Clustering of hDRG neurons by Conos. (**b**) The accuracy of neural-network classifier in learning hDRG neurons was visualized as learning curve. The Y-axis represents learning accuracy, and the X-axis represents the training epoch numbers. Following the training epochs, the maximum accuracy plateaus of this learning curve reaching ~88%. (**c**) Percentage bar chart visualization, the consistency of assigned hDRG neurons by the neural-network scoring module. Blue and red sections of the bars indicate ratio of cells within each type with and without, respectively, consistent assigned to itself. (**d**) hierarchical heatmap visualization of the probabilistic similarity across cell types. The color gradient depicts similarity score from high (yellow, 100% similarity) to low (dark blue, 0% similarity). (**e**) Percentage of each cell type in single-soma RNA-seq dataset, 1066 neurons total. Source data

We also independently analyzed the data using graph-based clustering (Conos^22^; Extended Data Fig. 2a) and a neural-network-based probabilistic scoring module^7,9^ (Extended Data Fig. 2b–d). Cluster structure revealed by Conos analysis reproduced the Seurat results. The accuracy score of Seurat clustering assignment by the learning module was near 90% (Extended Data Fig. 2b), confirming that most cells were accurately assigned to their corresponding clusters (Extended Data Fig. 2c). Thus, three independent analyses confirmed the robustness of the clustering and supported the cell type assignment. The percentage of each cell type in the 1,066 neurons was calculated (Extended Data Fig. 2e).

**Extended Data Fig. 3:**
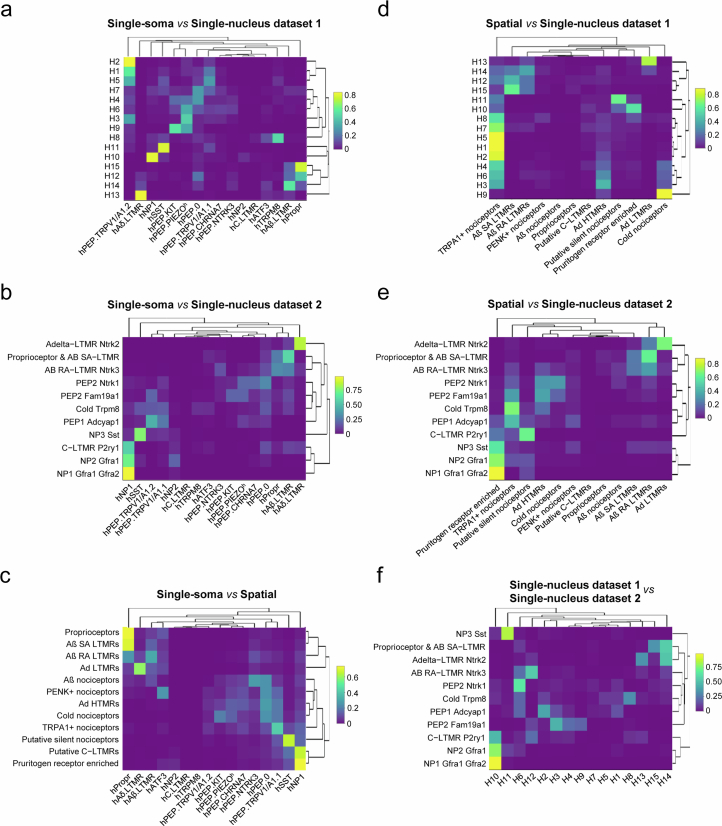
Correlation analysis of human DRG neuron cell types across our dataset and three published datasets. (**a-f**) Heatmaps comparing the similarities of human DRG neuron cell types between different datasets analyzed by Seurat Integration, Mapping and Annotating Query Datasets module. The color gradient represents cell type similarity. The maximum similarity is 1. Single-soma indicates the dataset of this study, single-nucleus dataset 1 indicates the Nguyen et al. dataset, single-nucleus dataset 2 indicates the Jung et al. dataset, and spatial indicates the Tavares-Ferreira et al dataset. Source data

Using Seurat integration, we performed cell type correlation analyses of our dataset and three other human DRG neuron sequencing datasets^12–14^ (Extended Data Fig. 3). Overall, the similarities across the different datasets were low. Our dataset correlated best with the Nguyen dataset^12^ (Extended Data Fig. 3a), with 4–5 highly corresponding cell types. NP1 in our dataset corresponded well with H10 in the Nguyen dataset, whereas hSST showed a strong one-to-one match across different datasets. Notably, our dataset is the only one that separated NP2 from NP1. Both our dataset and the Nguyen dataset^12^ identified two TRPM8^+^ populations: hTRPM8/H8 (TRPM8 high) and hPEP.KIT/H9 (TRPM8 low) (Extended Data Fig. 3a). The two other datasets identified only one TRPM8 cluster (Extended Data Fig. 3b–f). Aδ-LTMR assignments were consistent across datasets, whereas the clustering of other A-LTMRs and proprioceptors was more divergent. C-LTMR clusters were inconsistent across different datasets, and the correlations of PEP or nociceptor clusters among different datasets were generally weak. Our study identified seven PEP clusters, several of which did not have a strong correlation with clusters in other datasets (Extended Data Fig. 3a–c), implying that they might be previously unsegregated subtypes.

### Cross-species comparison of DRG neuron types

**Fig. 2:**
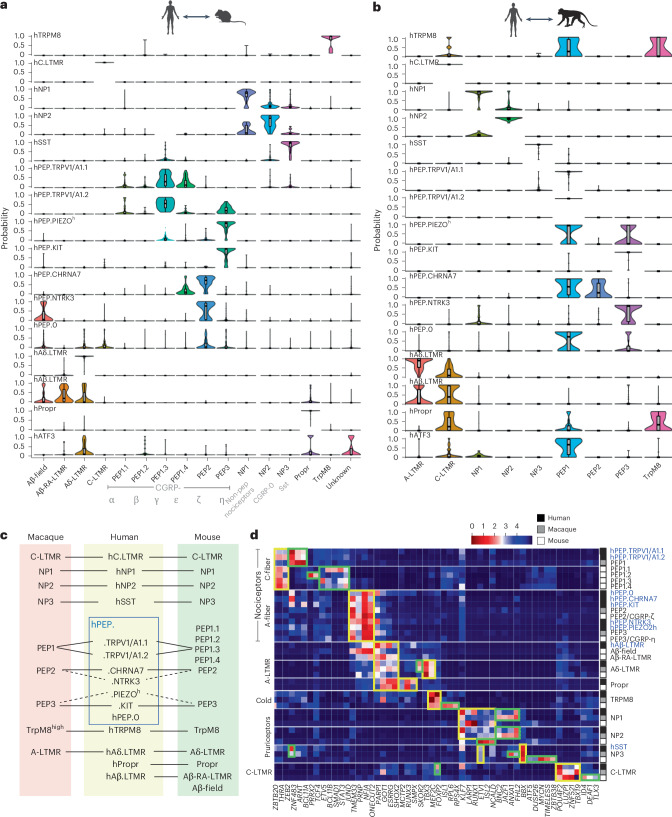
Cross-species analysis of DRG neurons in human, macaque and mouse. **a**,**b**, Conos label propagation from mouse^8^ (**a**, biological *n* = 6) and macaque^9^ (**b**, biological *n* = 5) to hDRG neuron (*n* = 6 DRGs obtained from *n* = 3 donors) clusters showing the cell type correlation. Mouse names combined Sharma^8^ and Usoskin^7^ nomenclature. For UMAPs of correspondent co-integration, from which these results were inferred, see Extended Data Fig. 4a,b. The lower and upper bounds of the box correspond to the first quartile (Q1, 25%) and the third quartile (Q3, 75%), respectively. A gray dot within the box represents the median value (Q2, 50%). The lower end of the whiskers indicates the minimal value within 1.5 times the interquartile range (IQR) from the first quartile (Q1, 25%). The upper end of the whiskers indicates the maximal value within 1.5 times the IQR from the third quartile (Q3, 75%). **c**, Summary of correspondence of DRG neuron clusters among three species. Solid lines depict clear match, and dashed lines represent partial similarity. **d**, Heatmap visualization of cross-species-conserved and species-specific TF-associated gene patterns across mouse, macaque and human. The color gradient bar depicts the distances of gene patterns from close (red) to distant (dark blue) in arbitrary units. Species are color coded in the right column. Yellow boxes, conserved GRNs; green boxes, species-specific GRNs. Non-pep, non-peptidergic. Source data

**Extended Data Fig. 4:**
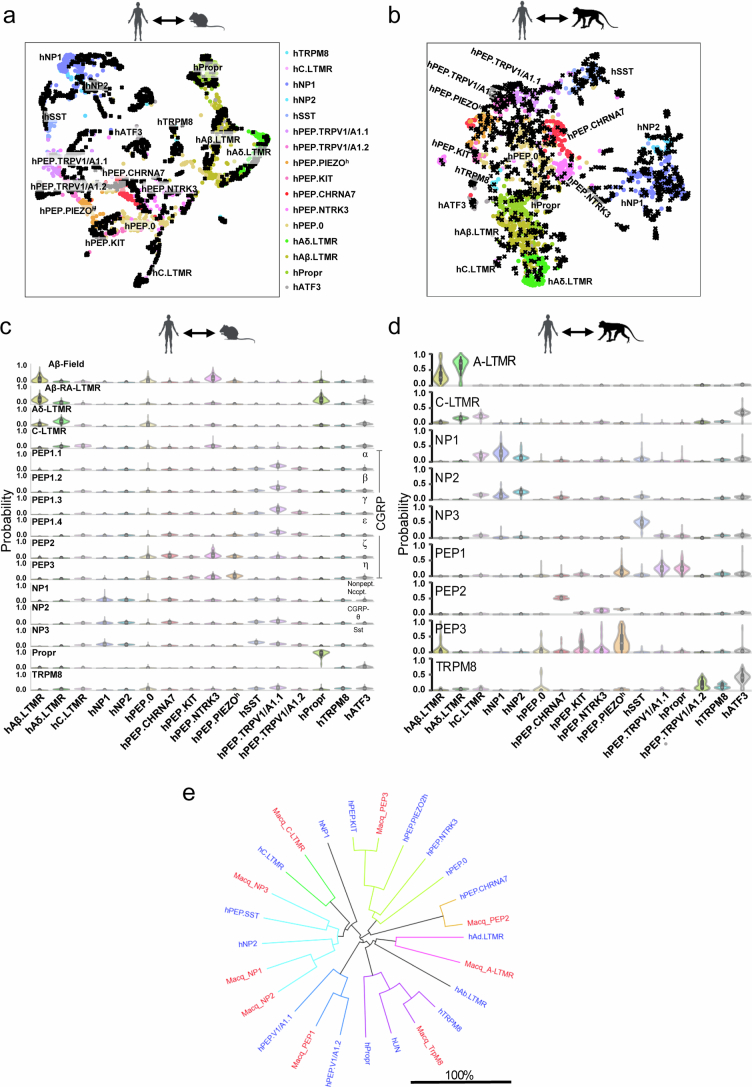
Cross-species comparison of sensory neuron types among human, macaque, and mouse. (**a-b**) UMAPs of Conos co-clustering of human and mouse (**a**) or macaque (**b**) DRG neurons. These co-clustering were used for label propagation inferences shown on Fig. 2a & b. Each pair UMAPs were plotted with the same coordinates, the human clusters are shown in color, the small black uncolored crosses depict mouse and macaque clusters. (**c-d**) Probabilistic neural network probability scores of mouse (Sharma dataset, biological N = 6) (**c**) and macaque (Kupari, SmartSeq2 dataset, biological N = 5) (**d**) DRG neuron types tested on human trained module (n = 6 DRGs obtained from N = 3 donors). The violin plot parameters are the same as the supplementary fig. 1. (**e**) Radial hierarchical dendrogram showing the similarity between cell types in humans (**h**, blue) and macaques (Macq_, red). Branch lengths indicate the degree of similarity. The scale bar from center to the terminus represents 100% similarity. Source data

We performed a cross-species comparison among our human dataset, a mouse dataset^8^ and a macaque dataset (SmartSeq2 dataset)^9^. We used three different strategies: Conos pairwise co-clustering followed by label propagation (Fig. 2a,b and Extended Data Fig. 4a,b); probabilistic neural network learning (Extended Data Fig. 4c,d); and machine-learning-based hierarchical clustering of an integrated dataset of human and macaque (Extended Data Fig. 4e). In all these analyses, human non-PEP DRG neuron types showed a high correlation with those of mouse and macaque, including hC.LTMR, hNP1, hNP2, hSST (NP3 in mouse and macaque), hTRPM8, hAδ.LTMR, hAβ.LTMR and hPropr neurons (Fig. 2a,b and Extended Data Fig. 4). hPEP.TRPV1/A1.1 and hPEP.TRPV1/A1.2 corresponded to macaque PEP1 (ref. ^9^) and mouse subclass PEP1.3/CGRP-γ, suggesting that these clusters represent C-fiber thermoreceptors and nociceptors. Notably, there are four types of mouse C-fiber PEP (CGRP) nociceptors^9,23^, but our analysis indicated that mouse PEP1.1/CGRP-α, PEP1.2/CGRP-β and PEP1.4/CGRP-ε had low homology to human PEP C-fibers (Fig. 2c). hPEP.CHRNA7 showed a high correlation with mouse PEP2/CGRP-ζ and macaque PEP2, and hPEP.KIT corresponded to mouse PEP3/CGRP-η and macaque PEP3 (Fig. 2c). hPEP.NTRK3, hPEP.PIEZO^h^ and hPEP.0 lacked one-to-one counterparts in mouse or macaque (Fig. 2c). hPEP.NTRK3 might represent a convergent mouse PEP2/3-like cell type, whereas hPEP.PIEZO^h^ showed some similarity to mouse PEP3 (CGRP-η) and macaque PEP1 and PEP3. hPEP.0, a type of human PEP A-afferent, showed no similarity to mouse DRG neurons but some relationship to macaque PEP1 and PEP3, suggesting that it might be a primate-specific PEP nociceptor.

Transcription factors (TFs) play a critical role in DRG neuron development and differentiation^24^. Thus, we performed a TF-associated gene regulatory network (TF-GRN) analysis (Fig. 2d). We observed evolutionarily conserved TF-GRNs defining C-fiber nociceptors, A-fiber nociceptors, A-LTMRs, TRPM8, C-fiber pruriceptors/nociceptors (hNP1, hNP2 and hSST) and C-LTMRs (yellow boxes) as well as species-specific networks, such as for C-fiber nociceptors, hTRPM8, hNP1, hNP2, hSST and hC.LTMR (green boxes).

### Top marker genes comparison across species

**Fig. 3:**
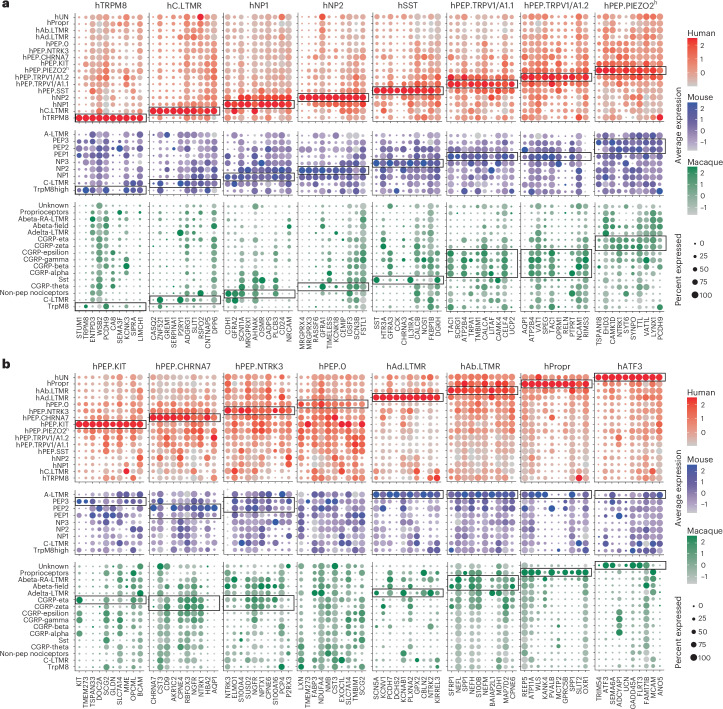
Comparison of marker gene expression across species. **a**,**b**, Dot plots showing top 10 specific marker genes selected from each hDRG neuron cluster expressed in human (red), macaque^9^ (blue) and mouse^8^ (green) DRG neuron datasets. The color scale represents the average expression level (log-normalized counts) of each gene in the clusters. The black boxes highlight the corresponding cell types based on the label transfer and neural network scoring analysis. Non-pep, non-peptidergic. Source data

We selected the top 10 marker genes from each hDRG neuron population and compared them to the corresponding cell types in macaque^9^ and mouse^8^ (Fig. 3a,b). In general, these gene expression patterns were more similar between human and macaque than between human and mouse. Some genes were expressed in the corresponding populations across all three species. For example, *TRPM8* was expressed in C-fiber cold-sensing neurons, and *IL31RA* was expressed in the nociceptive/pruriceptive population (NP3) and human corresponding cluster hSST. Some genes, such as *KCNV1*, were specific to primate DRG neurons, and some marker genes, such as *STUM*, were specific to hDRG neurons. An interactive web resource for browsing the dataset is available at https://ernforsluolabs.shinyapps.io/HumanDRG/.

### Molecular marker expression and validation

**Fig. 4:**
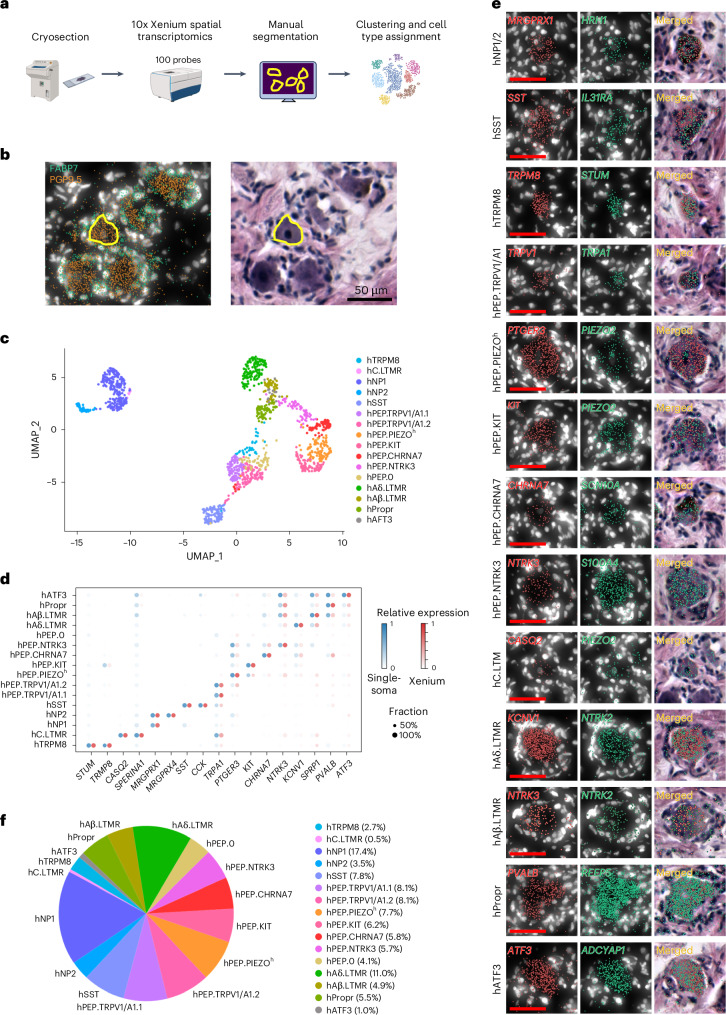
10x Xenium spatial transcriptomics of hDRG neurons. **a**, Overall workflow of 10x Xenium spatial transcriptomics and data analysis. **b**, Representative images of *PGP9.5* (*UCHL1*, orange)/*FABP7* (green) and H&E staining used for manual segmentation (the experiment was repeated independently four times with similar results). **c**, UMAP plot showing the cluster annotation of 1,340 hDRG neurons from 10x Xenium spatial transcriptomics. Four DRG sections from two donors (section 1, donor N3 L2; section 2, donor N4 L3; section 3, donor N4 T12; section 4, donor N4 T12) were used. **d**, Dot plot showing the top marker genes expressed in each hDRG neuron cell type from single-soma sequencing (blue) and Xenium (red). The color scale represents the normalized average expression level (from 0 to 1) of each gene in the clusters. **e**, Example images showing expression of marker genes expressed in each cell type (scale bar, 50 μm) (the experiment was repeated independently four times with similar results). **f**, Percentage of each cell type in the 10x Xenium dataset. Source data

**Extended Data Fig. 5:**
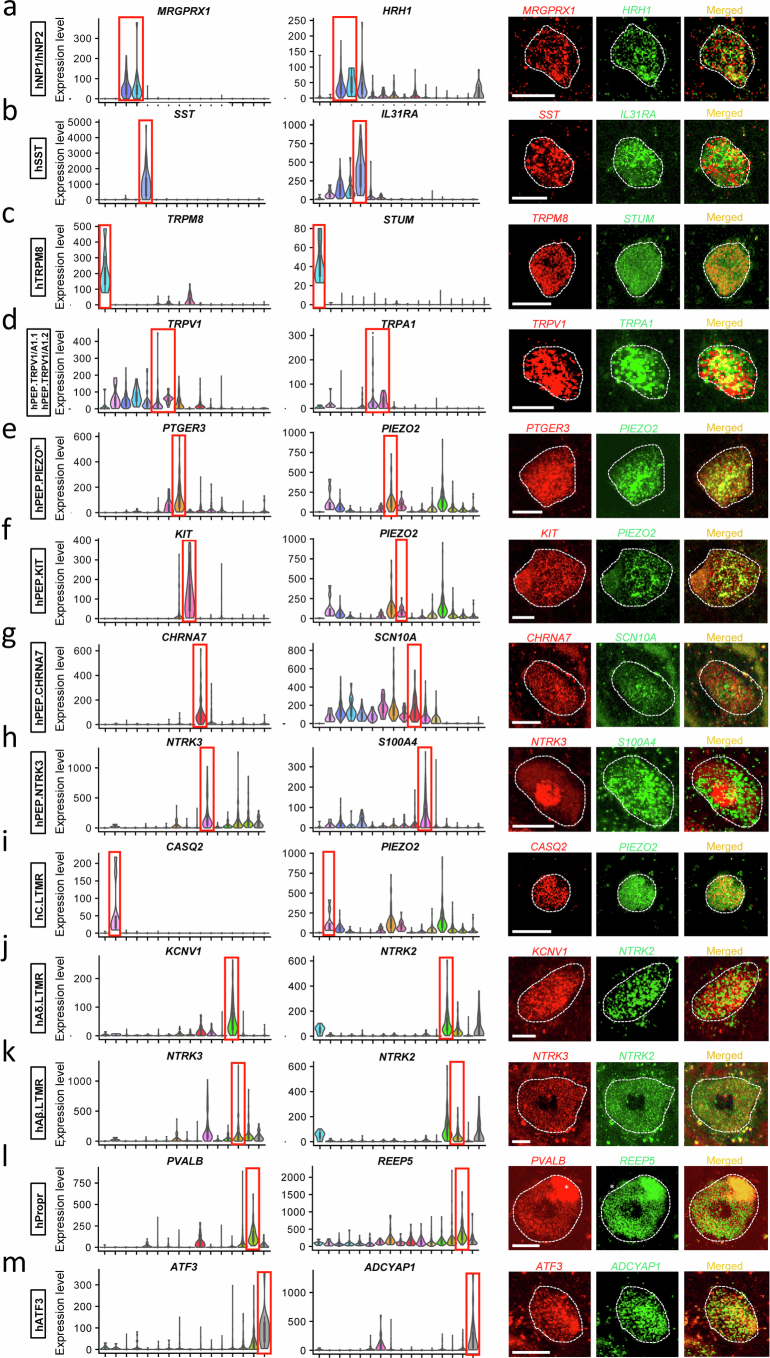
Marker gene expression and validation in human DRG neurons by RNAScope in situ hybridization. (**a**-**m**) Marker genes selected for labeling of different clusters (left) and validation by multiplex FISH (middle). The fluorescent images show detected transcripts in one example hDRG neuron (cell body outlined by the white dashed line. These experiments were repeated with DRG sections from two donors (donor N3, lumbar DRG L2; donor N4, thoracic DRG T12). The violin plot parameters are the same as the Extended Data Fig. 1. Scale bar: 25 μm. Source data

We validated the expression of specific marker genes using RNAscope multiplex fluorescence in situ hybridization (FISH) (Extended Data Fig. 5a–m) and 10x Xenium spatial transcriptomics (Fig. 4). One hundred marker genes from our single-soma sequencing dataset (including 87 neuronal genes and 13 non-neuronal genes) were selected for 10x Xenium spatial transcriptomics (Fig. 4a). We performed manual segmentation based on expressions of pan-neuronal marker gene PGP9.5, satellite glia cell marker FABP7 and the corresponding hematoxylin and eosin (H&E) staining (Fig. 4b). In total, 1,340 neurons were analyzed and clustered (Fig. 4c). Sixteen clusters were identified and assigned as different cell types according to marker gene expression, which showed a similar pattern between Xenium and single-soma cell types (Fig. 4d). The 10x Xenium spatial transcriptomics results validated the top marker gene expression (Fig. 4e) and cell type classification of our single-soma dataset. The proportion of each cell type in the Xenium dataset was calculated (Fig. 4f).

**Fig. 5:**
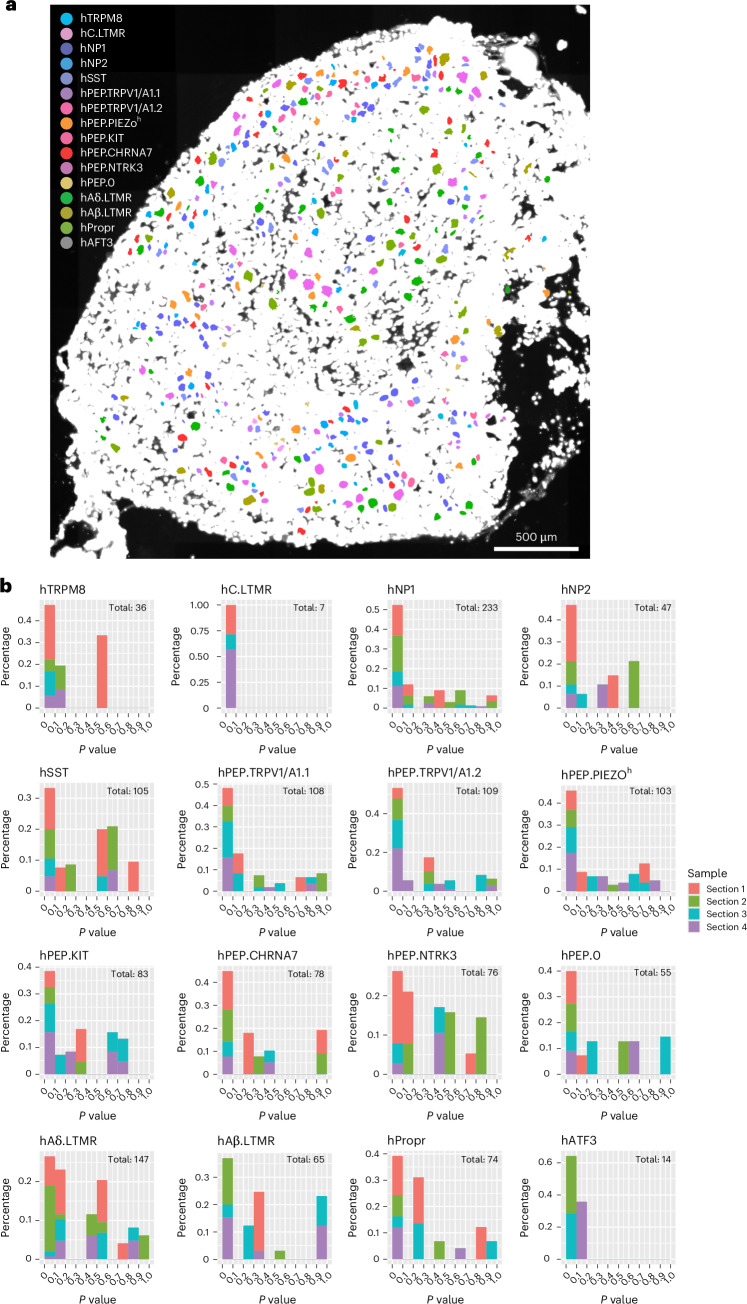
Spatial distribution of hDRG neurons. **a**, Spatial distribution of different types of DRG neurons in one example hDRG section (scale bar, 500 μm). **b**, Clustering probability of each neuron within a given type. Pearson’s chi-squared test with degree of freedom of 1 was used, and no adjustments for multiple comparisons were made for the data analysis. *x* axis is the *P* value, and *y* axis is the frequency (percentage of cell count). Different colors indicate data from different hDRG sections (section 1, donor N3 L2; section 2, donor N4 L3; section 3, donor N4 T12; section 4, donor N4 T12). Low *P* value (*P* < 0.05) indicates significant cell clustering. Source data

The spatial distribution of each hDRG neuron soma with its assigned cell type was visualized (Fig. 5a). Different hDRG neuron types were intermingled in a DRG section without obvious spatial segregation. However, for neurons within each type, cell clustering probability analysis (calculating *P* value by comparing the observed density to the theoretical even-distribution density in a cell-centered 500-µm-diameter circle; Fig. 5b) revealed spatial clustering. More than 40% of cells in hTRPM8, hC.LTMR, hNP1, hNP2, hPEP.TRPV1/A1.1, hPEP.TRPV1/A1.2, hPEP.PIEZO^h^, hPEP.CHRNA7 and hATF3 populations showed significant clustering (*P* < 0.05).

### Conjecturing function based on molecular features

**Extended Data Fig. 6:**
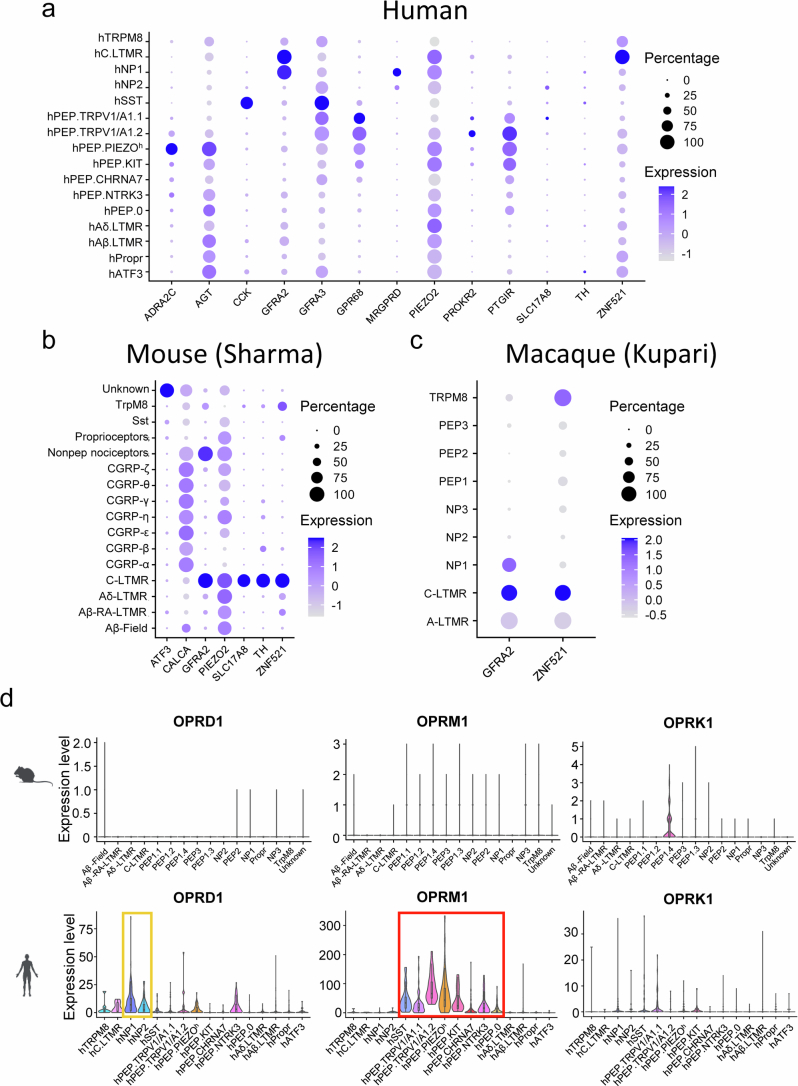
Expression of additional marker or functional genes in human, mouse and macaque DRG neuron cell types. (**a-c**) Dot plots showing the expression of additional marker or functional genes in human (**a**), mouse (**b**) and macaque (**c**) DRG neuron cell types. The color scale represents the log-normalized expression levels of each gene across cells. (**d**) Violin plot showing the expression of opioid receptors in mouse and human DRG neurons. The violin plot parameters are the same as the Extended Data Fig. 1. Source data

hNP1 and hNP2 exclusively expressed *MRGPRX1* (Fig. 4d,e), a pruritogen-activated Mas-related GPCR family member^25^, and highly expressed *HRH1* (Fig. 4e), indicating that these two populations could be activated by various pruritogens. *MRGPRX4* (a bile acid sensing itch GPCR) was enriched in hNP2 but not hNP1 (Fig. 4d), whereas *PIEZO2* was expressed at a higher level in hNP1 than in hNP2 neurons. In mice, NP1 neurons highly express *Mrgprd*, and NP2 neurons express *MrgprA3* (ref. ^7^). In humans, however, *MRGPRD* was expressed in only a few NP1 neurons (Extended Data Fig. 6a), and the *MRGPRA3* gene does not have a human orthologue. Thus, hNP1 and hNP2 populations likely have conserved functions in mediating itch sensation but involve different key molecular receptors.

The hSST population displayed specific expression of *SST* and an enriched expression of *GFRA3* and *CCK* (Fig. 4d,e and Extended Data Fig. 6a). This cluster corresponded to the mouse and macaque NP3 populations^7,8^. Given the role of mouse NP3 neurons in itch sensation^26^ and the high expression of receptors, such as *HRH1* and *IL31RA*, hSST afferents could also mediate itch and inflammation^7^. hSST neurons also co-expressed the peptidergic neuron marker *CALCA*, which was barely detected in the corresponding mouse NP3 neurons (Extended Data Fig. 6b)^8,9^.

The hTRPM8 population was distinguished from other cell types by high-level expression of TRPM8 and the specific expression of *STUM* (Fig. 4d,e). Some hTRPM8 neurons expressed low levels of *TRPV1*, suggesting that these neurons might also be activated by heat stimuli. Consistently, human physiological recordings identify cold-sensitive C-fibers responding to heating^27^.

The two peptidergic C-fiber clusters hPEP.TRPV1/A1.1 and hPEP.TRPV1/A1.2 displayed overlapping high expression of *TRPV1* and *TRPA1* (Fig. 4e). Because *TRPV1* is activated by noxious heat and capsaicin, and *TRPA1* is activated by various noxious chemicals^28,29^, these populations are likely C-fiber peptidergic thermoreceptors and nociceptors. hPEP.TRPV1/A1.2 neurons expressed genes enriched in mouse viscera-innervating DRG neurons^30^, such as PROKR2 and PTGIR (Extended Data Fig. 6a), suggesting that this population might innervate deep tissues.

The hPEP.PIEZO^h^ cluster expressed *PIEZO2* transcripts at a level similar to hC.LTMR and hA.LTMRs. *ADRA2C*, which was mainly detected in hPEP.PIEZO^h^ (Extended Data Fig. 6a), is a specific adrenoreceptor in human sensory fibers innervating blood vessels^31^. In addition, this cluster expressed *GPR68*, a membrane receptor sensing flow and shear forces in the vascular endothelia cells^32^, and angiotensin (AGT), a peptide hormone that regulates blood pressure^33^ (Extended Data Fig. 6a). Moreover, these neurons expressed a high level of *PTGIR* (Extended Data Fig. 6a). Mouse *PTGIR*^+^ DRG neurons innervate the bladder^34^, and *PIEZO2* is required for bladder mechanical sensation^35^. Therefore, hPEP.PIEZO^h^ neurons might function in sensing mechanical forces from blood vessels and internal organs. Given that there is no clear mouse DRG PEP^+^ population with high *PIEZO2* expression, we speculate that hPEP.PIEZO^h^ neurons are human/primate specific or greatly expanded in humans/primates.

The hPEP.KIT cluster had specific expression of *KIT* and medium-to-low expression of *PIEZO2* (Fig. 4e), while in mouse DRG neurons, *Kit* is more broadly expressed and found in four peptidergic clusters (Fig. 3b). Cross-species analysis suggested that this cluster mainly corresponds to the PEP3 population in mouse and macaque, which are A-fiber high-threshold mechanoreceptors (HTMRs). Thus, the hPEP.KIT cluster likely functions as fast-conducting mechano-nociceptors.

The third peptidergic A-fiber cluster, hPEP.CHRNA7, featured abundant expression of *CHRNA7* but almost no expression of *PIEZO2* (Fig. 4e) and corresponded to the PEP2 populations in mouse and macaque. Interestingly, this cluster also expressed *PVALB*, a common molecular marker for proprioceptors. Retrograde tracing from the mouse gastrocnemius muscle labeled *CHRNA7*^+^ DRG neurons^34^. Thus, it is possible that hPEP.CHRNA7 may contain muscle-innervating A-fiber nociceptive sensory afferents.

hPEP.NTRK3 is a population of peptidergic A-fibers with high expression of *NTRK3* and *S100A4* and low expression of *PIEZO2* (Fig. 4e and Extended Data Fig. 5h,i). hPEP0 is a population of peptidergic A-fibers that expressed *CALCA* and a moderate level of *PIEZO2* but lacked other specific marker genes. Potential functions of hPEP.NTRK3 and hPEP0 are currently unclear. They could be some types of A-fiber mechano-nociceptors^36^.

hC.LTMR is the putative human C-tactile nerve fiber. Its specific marker gene, *CASQ2*, is not detected in either mouse or macaque C-LTMR (Fig. 3a). Conversely, specific molecular markers of mouse C.LTMR, *TH* and *SLC17A8* (*VGLUT3*) (Extended Data Fig. 6b), were barely detected in hDRG neurons (Extended Data Fig. 6a). Human, mouse and macaque C.LTMRs all had conserved expression of GFRA2 and zinc finger transcription factor ZNF521 (Extended Data Fig. 6a–c). hC.LTMR likely mediates innocuous affective touch sensation^37^.

A-LTMRs were characterized by large-diameter somata, high expression of *NTRK2* and *NTRK3* and lack of *SCN10A* and *PRDM12*. We identified four clusters in the A-LTMR category. (1) hAδ.LTMR was named based on its high expression level of *NTRK2* and *PIEZO2* but low levels of *NTRK3*. *KCNV1* was enriched in this cluster (Fig. 4d,e). (2) hAβ.LTMR expressed higher levels of *NTRK3* and lower levels of *NTRK2* compared to hAδ.LTMRs (Fig. 4d,e). (3) hPropr expressed high levels of *PVALB* and *REEP5* (Fig. 4d,e and Extended Data Fig. 5l). (4) hATF3 (Fig. 4d,e) contained mainly large-diameter neurons and corresponded strongly to the ‘unknown’ cluster identified in normal male mice^8^ (Extended Data Fig. 6b). ATF3 is a TF associated with sensory afferent injury. Because there were no medical records indicating obvious nerve injuries in our human donors, and the mouse data came from naive mice, we speculate that these neurons might represent a cluster that has undergone low levels of afferent insult, although we cannot exclude the possibility that it represents a population of normal DRG neurons.

### Insights for drug target discovery

Unlike mouse DRG neurons, which do not display cell-type-enriched expression patterns of opioid receptors (Extended Data Fig. 6d), we found that *OPRM1* was enriched in all hPEP clusters, whereas the δ-opioid receptor (*OPRD1*) was preferentially expressed in itch populations hNP1 and hNP2 (Extended Data Fig. 6d). Because opioid receptors are inhibitory GPCRs, these results suggest that activation of *OPRM1* could directly inhibit human nociceptive afferents, whereas *OPRD1* could be a molecular target for inhibiting itch transmission. On the other hand, *OPRK1* was barely detected in our dataset, suggesting that *OPRK1* agonists may relieve itch through indirect or other mechanisms.

**Extended Data Fig. 7:**
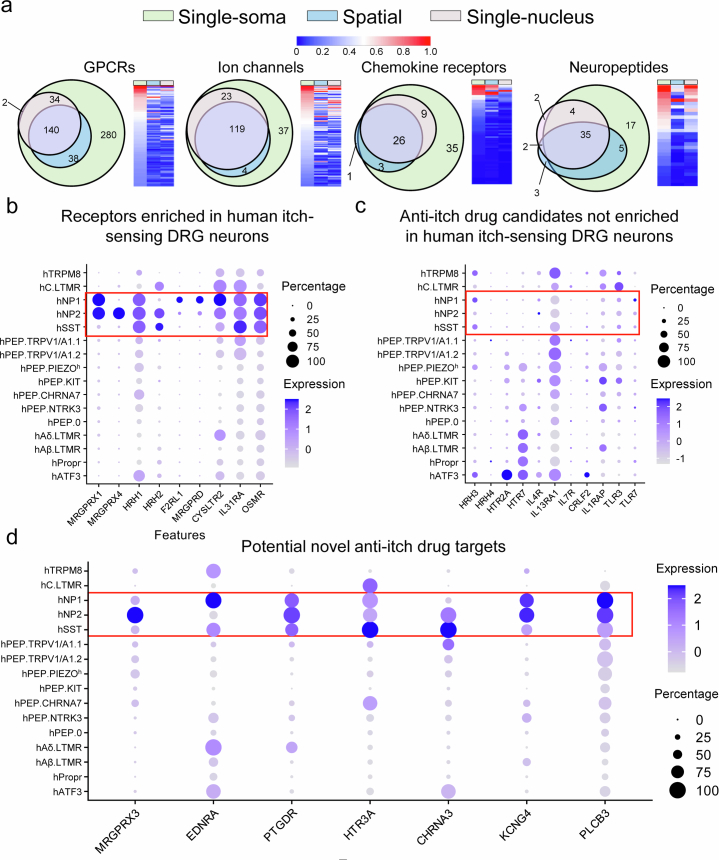
Single-soma deep RNA-seq dataset is powerful for molecular discovery. (**a**) Comparison of the number of GPCRs, ion channels, chemokine receptors, and neuropeptides in single-soma, spatial, and single-nucleus RNA-seq datasets. “Single-soma” is the dataset from this study, “Spatial” refers to the dataset from the Tavares-Ferreira et al, and “Single-nucleus” refers to the dataset from the Nguyen et al. The circles represent the number of detected genes in these gene families across the three datasets. The heatmaps display the expression levels of the top genes from each of the three datasets. The color scale represents the expression level normalized to the maximum gene expression in each dataset. (**b**-**c**) Expression of putative itch receptors in hDRG neurons shown by dot plots. Some receptors are highly enriched (**b**), while some receptors are barely detected (**c**) in human itch populations, which are highlighted by red boxes. (**d**) Novel GPCRs, ion channels, and other genes enriched in human itch populations shown by dot plot. For dot plots in panel **b**-**d**, the size of the dot represents the percentage of cells within a cluster, and the color corresponds to the average expression (scaled data) across all cells within a cluster for each gene shown. Source data

**Extended Data Fig. 8:**
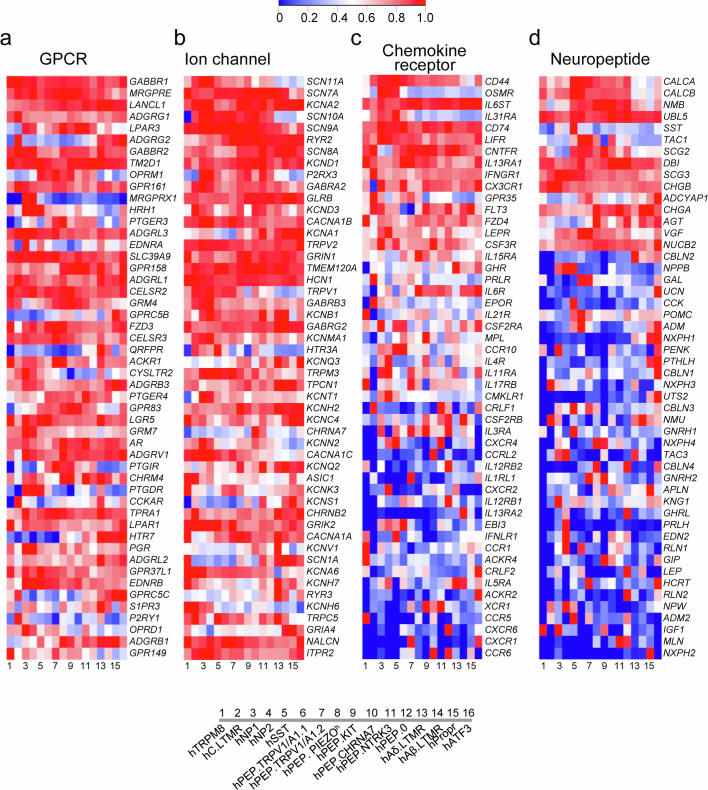
Expression of drug targets in hDRG neurons. (**a-d**) Heatmap showing the expression of top 50 GPCRs (**a**), ion channels (**b**), chemokine receptors (**c**) and neuropeptides (**d**) in hDRG neurons. Genes were ranked by average expression level in all DRG neurons. Color scale represents the average gene expression level normalized to the maximum average gene expression within each clusters. Source data

We identified a higher number of membrane proteins, such as GCPRs, ion channels, chemokine receptors and neuropeptides, compared to previous datasets (Extended Data Fig. 7a). Here, we analyzed itch-related receptor genes and molecules as an example. A subset of known itch receptors, such as *MRGPRX1*, *MRGPRX4* and *HRH1* (Extended Data Fig. 7b), was enriched in hNP1, hNP2 and hSST populations. However, some murine itch receptors were not enriched in human itch populations (Extended Data Fig. 7c). Instead, we identified other membrane receptors and signaling molecules (Extended Data Fig. 7d), such as MRGPRX3 and PTGDR, in human itch afferents. Top 50 GCPRs, ion channels, chemokine receptors and neuropeptides were also plotted (Extended Data Fig. 8).

### Immunostaining of sensory fibers in the human skin

**Extended Data Fig. 9:**
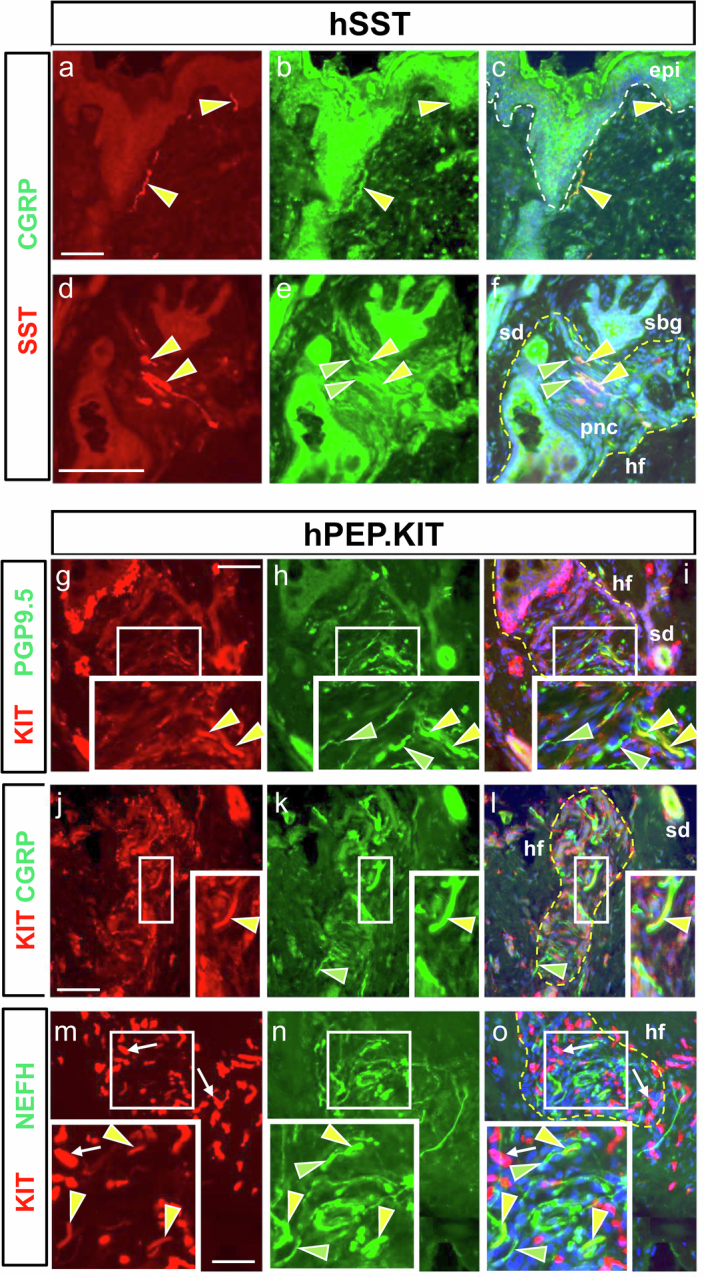
Immunostaining of different types of sensory fibers in the human skin using molecular makers identified by single-soma deep RNA-seq dataset. (**a-f**) Double immunostaining of human skin sections showing SST+ sensory fibers are a subset of CGRP+ sensory fibers in the dermis, the dermis-epidermis junction, and entering the epidermis (**a**-**c**) and near the hair follicle (**d**-**f**). The white dashed line indicates the dermis-epidermis junction. Yellow arrows indicate SST + /CGRP+ sensory afferents, green arrows indicate CGRP+ only sensory afferents (**g**-**o**). Double immunostaining of KIT with PGP9.5 (**g**-**i**), NEFH (**j**-**l**) and CGRP (**m**-**o**) in human hairy skin showing KIT+ sensory afferents are a subset of CGRP + /NEFH+ sensory afferents around the hair follicles. Inserts in large white rectangles are 2X enlargements of areas in the small white rectangles. Yellow dashed lines outline hair follicles. Yellow arrows indicate double positive sensory afferents, green arrows indicate PGP9.5+ only or CGRP+ only or NEFH+ only sensory afferents. In **m**–**o**, white arrows indicate macrophages that were previously known to be KIT + . Given that the macrophage KIT immunolabeling is far more intense than that on the innervation, the macrophages are overexposed and blurred at the exposure setting needed to capture the labeling of the innervation. The experiment was repeated independently three times with similar results. Epi, epidermis; hf, hair follicle; sd, sweat gland duct; pnc, pilo-neural complex; sbg, sebaceous gland. Scale bar, 100 μm. Source data

Although human afferents can be recognized by the pan-neuronal marker PGP9.5, different types of human sensory afferents are difficult to distinguish. Here, we conducted sensory afferent immunostaining using molecular markers identified by our dataset with leg skin biopsies from three healthy adult donors (Supplementary Table 3). *SST* is specifically expressed in hSST neurons, and we found SST^+^ sensory fibers in the dermis, the epidermis–dermis junction regions (Extended Data Fig. 9a-c) and near the hair follicle (Extended Data Fig. 9d–f). In addition, SST^+^ sensory fibers made up a subset of CGRP^*+*^ (encoded by *CALCA*) sensory afferents, confirming that hSST afferents had both CGRP transcripts and proteins. hPEP.KIT neurons specifically expressed *KIT* transcripts. A few KIT^+^ and PGP9.5^+^ sensory fibers were observed around hair follicles (Extended Data Fig. 9g–i). Consistent with RNA-seq results, KIT^+^ sensory fibers were also CGRP^+^ and NEFH^+^ (Extended Data Fig. 9j–o). In short, we were able to identify cutaneous hSST and hPEP.KIT afferents, demonstrating that our dataset is useful for selecting specific molecular markers to label different types of somatosensory afferents.

### Molecular-profile-informed microneurography recordings

**Fig. 6:**
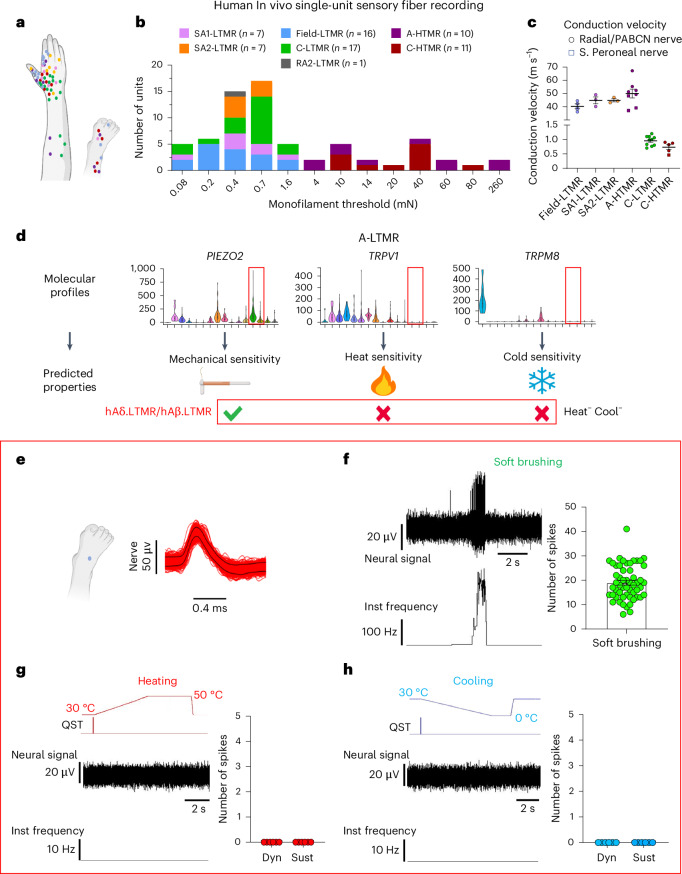
Molecular-profile-informed single-unit microneurography recordings of human skin afferents. **a**, Receptive field locations of single afferents from superficial peroneal (S. peroneal), posterior antebrachial cutaneous (PABCN) and radial nerve recordings (*n* = 69). All units are color coded and presented as filled circles, except for RA2-LTMR, which is shown without filling to indicate its location on the glabrous side of digit 1. Data were collected from 62 healthy participants (29 males and 33 females, 19–41 years of age). **b**, Distribution of mechanical (monofilament) thresholds for HTMRs and LTMRs in the recorded samples. **c**, Individual and mean (± s.e.m.) conduction velocities of different HTMR and LTMR types in response to surface electrical or mechanical stimulation (Field-LTMR: 40.4 ± 1.9 m s^−1^, *n* = 4 units; SA1-LTMR: 44.9 ± 2.6 m s^−1^, *n* = 3 units; SA2-LTMR: 44.9 ± 1.2 m s^−1^, *n* = 3 units; A-HTMR: 50.0 ± 3.3 m s^−1^, *n* = 8 units; C-LTMR: 1.0 ± 0.05 m s^−1^, *n* = 10 units; C-HTMR: 0.7 ± 0.08 m s^−1^, *n* = 5 units). **d**, Temperature sensitivity of hA.LTMR sensory fibers predicted based on gene expression obtained from the single-soma deep RNA-seq (the same violin plot parameters as in Fig. 1). **e**, Superimposed spike activity of an hA.LTMR field unit in response to repeated sensory stimulation of the receptive field. The receptive field location is indicated by the blue dot. **f**–**h**, Individual and mean (± s.e.m.) responses of hA.LTMR field units to soft brushing (**f**), heating (**g**, 4 °C per s) and cooling (**h**, 4 °C per s). The A-LTMR units responded robustly to soft brush stroking (*n* = 16 units, 54 trials, all displayed), with a mean ± s.e.m. of 19.0 ± 1.0 spikes. However, none of them responded to heating (*n* = 6 units, 17 trials) or cooling (*n* = 6 units, 15 trials). Each data point for heating and cooling (zero responses) represents at least two trials from an individual unit. Dyn, dynamic; Sust, sustained; Inst, instantaneous. Source data

Based on the expression patterns of heat/capsaicin-sensing TRPV1 and cold/menthol-sensing TRPM8 in hDRG neurons, we investigated the temperature/chemical sensitivity of five cutaneous afferent types. Single-unit axonal recordings (Fig. 6a) were performed from the radial, antebrachial and peroneal nerves of healthy participants using the microneurography technique. Mechanical thresholds, temperature sensitivity and conduction velocities of single afferents were measured (Fig. 6b,c).

Our sequencing dataset revealed a relatively high expression of *PIEZO2* and no *TRPV1* or *TRPM8* in human A-LTMRs (Fig. 6d). In 69 single-unit axonal recordings (Fig. 6a,b), we identified 16 Field-LTMR afferents^38^, a type of A-LTMR found abundantly in human hairy skin with characteristic low mechanical thresholds, exquisite sensitivity to soft brushing and fast conduction velocities (Fig. 6b,c,e,f). Consistent with the molecular predictions, they did not respond to cold or heat (Fig. 6g,h).

**Fig. 7:**
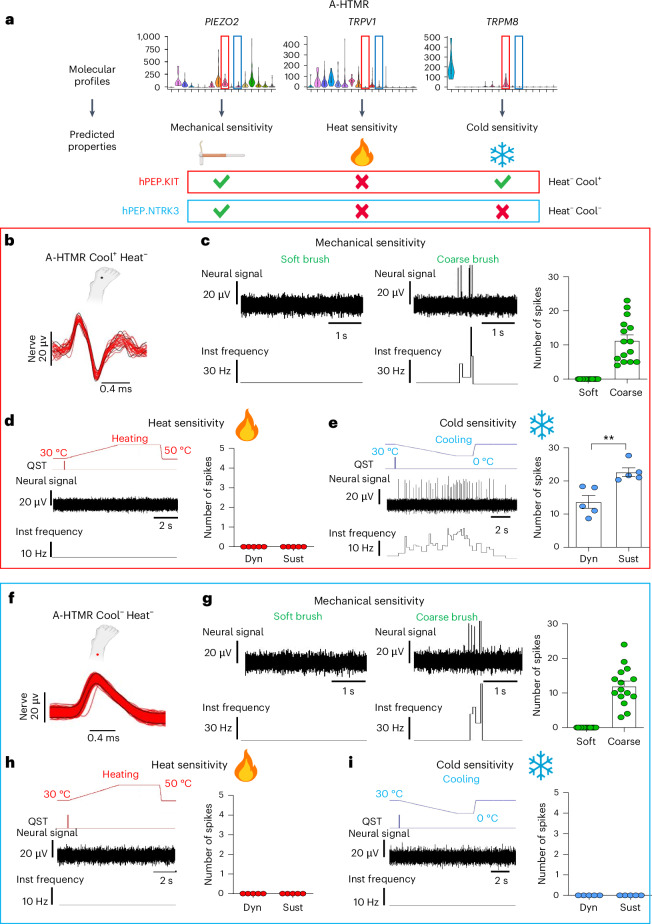
Molecular-profile-informed single-unit microneurography recordings of human A-HTMRs. **a**, Mechanical and temperature sensitivity of hA.HTMR sensory fibers predicted based on gene expression obtained from single-soma deep RNA-seq (the same violin plot parameters as in Fig. 1). **b**, Superimposed spike activity of an A-HTMR Cool^+^ Heat^−^ unit (putatively a hPEP.KIT neuron) in response to repeated sensory stimulation of the receptive field. The receptive field location is indicated by the blue dot. **c**–**e**, Individual and mean (± s.e.m.) responses of A-HTMR Cool^+^ Heat^−^ units to brushing (**c**), heating (**d**) and cooling (**e**). The A-HTMR Cool^+^ Heat^−^ units predictably showed no response to soft brushing, but all responded to coarse brushing (*n* = 5 units, each tested in triplicate, all trials shown). Furthermore, they did not respond to heating but displayed responses to cooling during both the dynamic (4 °C per s) and sustained phases. Each data point for heating and cooling represents the mean of triplicate responses from an individual unit (*n* = 5 units). An increased response was observed during sustained cooling compared to dynamic cooling (two-tailed paired *t*-test: *t*(4) = 4.889, *P* = 0.0081). **f**, Superimposed spike activity of an A-HTMR Cool^−^ Heat^−^ unit in response to mechanical stimulation of the receptive field. The receptive field location is indicated by the red dot. **g**–**i**, Individual and mean (± s.e.m.) responses of A-HTMR Cool^−^ Heat^−^ units to soft and coarse brushing (**g**), heating (**h**) and cooling (**i**). All A-HTMR Cool^−^ Heat^−^ units responded to coarse brushing, whereas none responded to soft brushing (*n* = 5 units, each tested in triplicate, all trials shown). Furthermore, none responded to heating or cooling. Each data point for heating and cooling represents a mean of triplicate responses from an individual unit (*n* = 5 units each). **P ≤ 0.01. Dyn, dynamic; Sust, sustained; Inst, instantaneous. Source data

**Extended Data Fig. 10:**
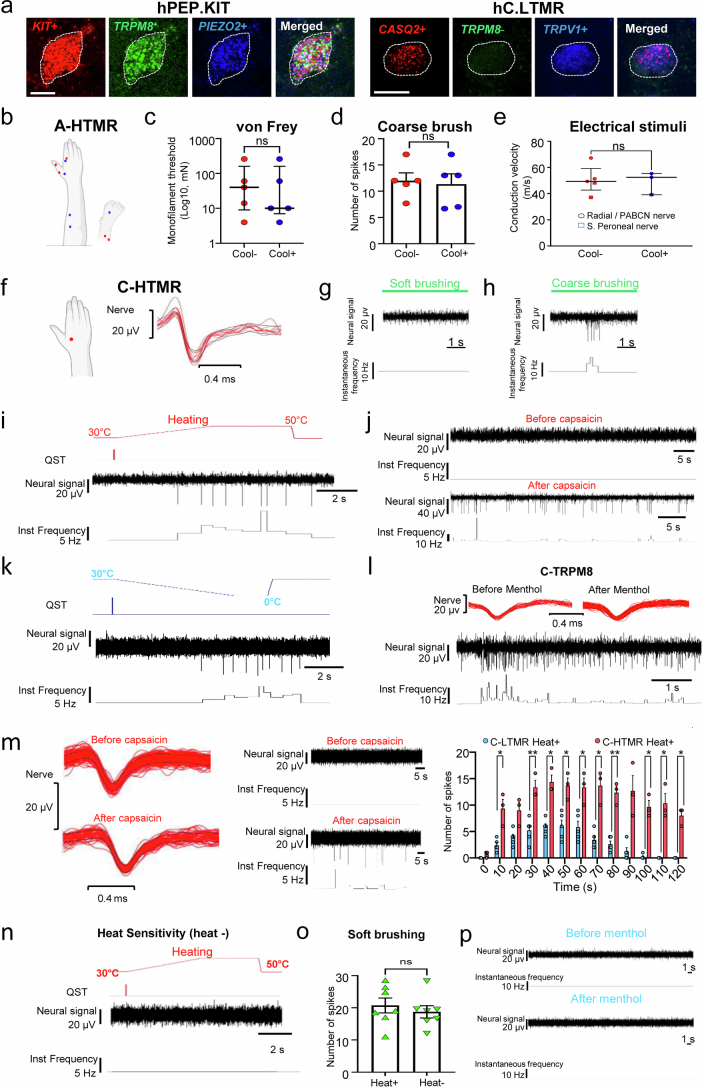
Molecular expression in the hPEP.KIT and hC.LTMR populations, and additional physiological characterization from human A-, C-HTMRs and C-LTMRs. (**a**) Validation of *TRPM8*, *PIEZO2*, *CGRP*, *CASQ2* and *TRPV1* expression in the hPEP.KIT and hC.LTMR populations by multiplex FISH (The experiment was repeated independently two times with similar results, the same violin plot parameters as the Fig. 1). Scale bar, 25 μm. (**b**) Receptive field locations of A-HTMR Cool+ and Cool- units. (**c**-**e**) Comparisons between A-HTMR subtypes showed no significant differences in monofilament thresholds (median ± IQR, n = 5 units each; Two-tailed independent, Mann-Whitney test: U = 10.50, P = 0.7619; panel **c**), coarse-brush responses (mean of triplicate responses per unit, pooled mean ± SEM, n = 5 units each; Two-tailed unpaired t-test: t(8) = 0.2692, P = 0.7946; panel **d**), and conduction velocities (median ± IQR, Cool- units: n = 5, Cool+ units: n = 3; Two-tailed independent Mann Whitney test: U = 6, P = 0.7857, panel **e**). (**f**) Superimposed spike activity of a C-HTMR Heat+ (MH) unit in response to repeated sensory stimulations of the receptive field. (**g**-**l**) Responses of a C-HTMR (MH) to soft-brushing (**g**), coarse-brushing (**h**), heating (**i**), and capsaicin (**j**). (**k**) Response of a C-HTMR (MC) to cooling. (**l**) Response of a TRPM8 (mechano-unresponsive C cold) unit to menthol. (**m**) Individual and mean ( ± SEM) responses of C-LTMR Heat+ and C-HTMR Heat+ units to capsaicin. Capsaicin to the receptive field evoked spontaneous activity in both C-HTMRs (n = 3 units) and C-LTMRs (n = 5 units). The effect was more robust (F (1, 6) = 150.1, P < 0.0001) and longer-lasting (F (2.695, 16.17) = 15.92, P < 0.0001) in C-HTMRs compared to C-LTMRs (2-way RM-ANOVA). Multiple comparisons were performed using Uncorrected Fisher’s LSD test. The duration of spontaneous activity in C-HTMRs is truncated to 120 s (post-capsaicin removal), aligning with the disappearance of spontaneous activity in C-LTMRs. (**n**) Example trace of a heat-unresponsive C-LTMR. (**o**) Soft-brush responses were not different between heat-responsive and non-heat-responsive C-LTMRs (mean of at least two responses per unit, pooled mean ± SEM, n = 7 units each; Two-tailed unpaired t-test: t(12) = 0.6649, P = 0.5187). (**p**) Example trace showing no response to menthol in a hC.LTMR. *P < 0.05, **P < 0.01. Source data

Human A-HTMRs are fast-conducting, high-threshold mechanoreceptors that rapidly mediate pain^36^. Our dataset suggested two potential molecular populations for cutaneous A-HTMRs: hPEP.KIT (Fig. 7a), corresponding to the mouse PEP3/CGRP-η cluster^7,9^ (Fig. 2a–c), a population of TRPV1^−^ fast-conducting hair-pull-sensitive mechano-nociceptors^39^, and hPEP.NTRK3 (Fig. 7a). In our dataset, hPEP.KIT neurons showed medium *TRPM8* expression (Fig. 7a), a feature not reported in mice. 10x Xenium spatial transcriptomics (Fig. 4d) and multiplex FISH (Extended Data Fig. 10a) validated the co-expression of *KIT*, *PIEZO2* and *TRPM8* in hDRG neurons. Conversely, hPEP.NTRK3 neurons expressed low levels of *PIEZO2* and were almost negative for *TRPV1* and *TRPM8* (Fig. 7a). During microneurography recordings, we identified 10 A-HTMRs (Fig. 6b and Extended Data Fig. 10b) based on insensitivity to soft brush stroking (while responding to a coarse brush), high indentation thresholds (≥4 mN; Figs. 6b and 7b,c,f,g) and Aβ-range conduction velocities (>30 m s^−1^; Fig. 6c). All A-HTMRs were heat insensitive (Fig. 7d,h), with half responding to cooling (*n* = 5) (Fig. 7e,i). Cool^+^ A-HTMRs responded to both dynamic and sustained phases (Fig. 7e). No significant differences were found between Cool^+^ and Cool^−^ A-HTMRs in mechanical thresholds, coarse brush responses or conduction velocities (Extended Data Fig. 10c–e).

**Fig. 8:**
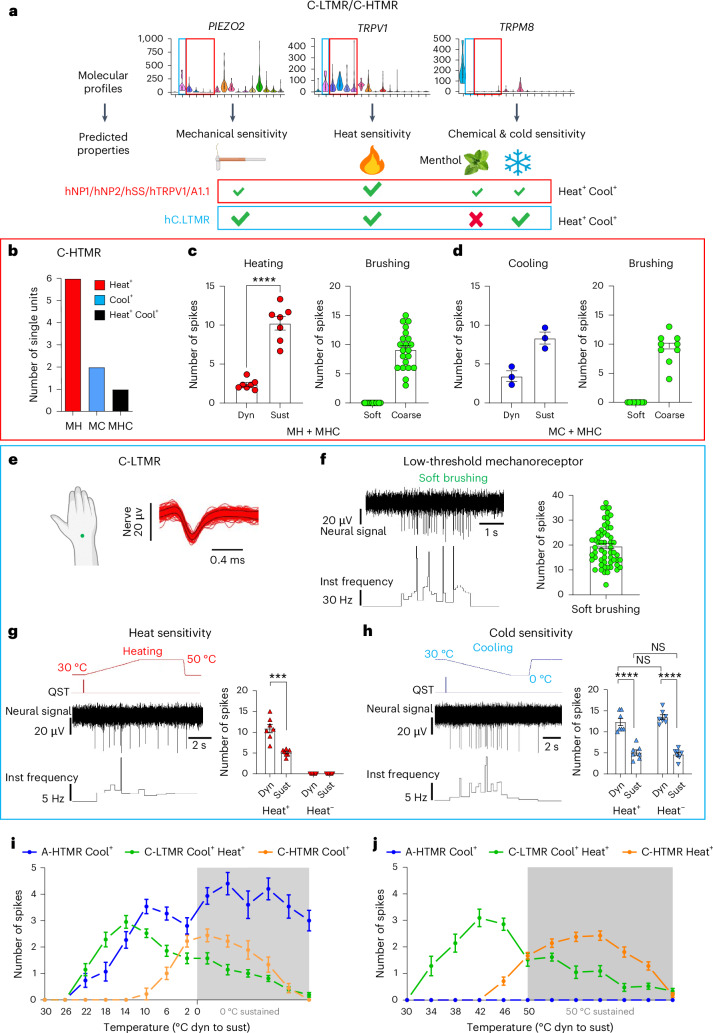
Molecular-profile-informed single-unit microneurography recordings of human C-HTMRs and C-LTMRs. **a**, Temperature sensitivity of hC-HTMRs and hC-LTMRs predicted based on gene expression from single-soma deep RNA-seq (the same violin plot parameters as in Fig. 1). **b**, Number of C-HTMRs responding to heating (MH), cooling (MC) and both (MHC). **c**,**d**, Individual and mean (± s.e.m.) responses of C-HTMRs to brushing (**c**,**d**), heating (**c**) and cooling (**d**). MH and MHC units showed no response to soft brushing but responded to coarse brushing (*n* = 7 units total, each tested in triplicate, all trials shown). Temperature data are shown as the mean of triplicate responses for each unit, with increased responses during sustained heating for MH and MHC units compared to dynamic heating (*n* = 7 units; two-tailed paired *t*-test: *t*(6) = 9.667, *P* < 0.0001). A similar pattern was observed for sustained cooling in MC and MHC units (*n* = 3 units total). **e**, Superimposed spike activity of an hC.LTMR in response to repeated sensory stimulation of the receptive field (marked by the green dot). **f**–**h**, Individual and mean (± s.e.m.) responses of hC.LTMRs to soft brushing (**f**), heating (**g**) and cooling (**h**). C-LTMRs responded robustly to soft brushing (*n* = 17 units, 57 trials, all displayed). Heating was tested in 14 C-LTMRs, with half responding. Each data point represents the mean of triplicate responses, with increased responses during dynamic heating in heat-responsive C-LTMRs compared to sustained heating (*n* = 7 units; two-tailed paired *t*-test: *t*(6) = 6.591, *P* = 0.0006). All 14 units responded to cooling. Each data point represents the mean of triplicate responses, with increased responses during dynamic cooling compared to sustained cooling (two-way ANOVA: *F*_1,24_ = 144.6, *P* < 0.0001; Tukey’s test: *P* < 0.0001). No significant differences (*P* > 0.05) were observed in cooling (**h**) between heat-responsive and non-heat-responsive C-LTMRs (two-way ANOVA: *F*_1,24_ = 0.2860, *P* = 0.5977). **i**,**j**, Spike counts for A-HTMRs, C-LTMRs and C-HTMRs in response to cooling (**i**) or heating (**j**) stimuli. Each data point represents the mean (± s.e.m.) responses of five A-HTMR Cool^+^, seven C-LTMR Cool^+^ Heat^+^, three C-HTMR MC/MHC and seven C-HTMR MH/MHC units, tested in triplicate. For C-afferent responses, conduction delays were adjusted based on the latency of electrically triggered spiking. ***P ≤ 0.001; ****P ≤ 0.0001. Dyn, dynamic; NS, not significant; Sust, sustained; Inst, instantaneous. Source data

Our recordings also identified cutaneous C-HTMRs (*n* = 11), likely corresponding to hNP1, hNP2, hSST and hPEP.TRPV1/A1.1 (Fig. 8a). These populations shared high *TRPV1* expression, with only some hPEP.TRPV1/A1.1 neurons expressing low levels of *TRPM8*. C-HTMR afferents responded to course brush stroking but not soft brush stroking, had high indentation thresholds (≥10 mN) and had slow conduction velocities (~1 m s^−1^; Fig. 6b,c and Extended Data Fig. 10f–h). They were further classified into mechano-heat (MH), mechano-cold (MC) and mechano-heat-cold (MHC) subtypes (Fig. 8b). As predicted, most C-HTMRs responded to heating/capsaicin (Fig. 8c and Extended Data Fig. 10i,j), whereas only a small subset responded to cooling (Fig. 8b,d and Extended Data Fig. 10k).

hC.LTMRs expressed *TRPV1* but not *TRPM8* (Fig. 8a), with RNAscope confirming *CASQ2*^+^ hC.LTMRs as TRPV1^+^ and TRPM8^−^ (Extended Data Fig. 10a). This pattern suggested that hC.LTMRs may respond to heating and capsaicin, a property not observed in rodents and non-human primates. C-LTMRs (*n* = 17) were identified by their soft brush sensitivity, low indentation threshold (≤1.6 mN) and slow conduction velocity (~1 m s^−1^, *n* = 10; Figs. 6a–c and 8e,f). During microneurography recordings, a subset of C-LTMRs responded to dynamic heating (7 out of 14 units; Fig. 8g). In five Heat^+^ C-LTMRs with stable recordings, we applied capsaicin to their receptive fields. Consistent with the TRPV1 expression, all five were activated by capsaicin (Extended Data Fig. 10m). Although human (Fig. 8a) and mouse C-LTMRs have almost no expression of *TRPM8* (ref. ^8^), they responded to cooling (Fig. 8h)^40–42^. No significant differences were found between Heat^+^ and Heat^−^ C-LTMRs in cooling and soft brush responses (Fig. 8h and Extended Data Fig. 10n,o). The response to temperature changes, although modest compared to mechanical stimulation, was reproducible, confirmed in triplicates for each modality and recording. As predicted, C-LTMRs showed no response to menthol^43^ (Extended Data Fig. 10p), unlike an hTRPM8 unit (mechano-unresponsive C-cold), which responded robustly to menthol (Extended Data Fig. 10l). These findings demonstrate that human C-LTMRs have polymodal response properties.

In comparing heating and cooling responses (Fig. 8i,j), A-HTMR Cool^+^ fibers responded to both dynamic and sustained cooling, whereas the responses of C-LTMRs and C-HTMRs tapered off during sustained thermal stimulation. C-HTMRs preferred noxious temperatures, whereas C-LTMRs preferred innocuous temperatures. This suggests that C-LTMRs may be a polymodal channel for innocuous or pleasurable touch, cooling and warmth.

## Discussion

A major challenge in conducting single-cell RNA-seq of human DRG neurons is separating sensory neurons from the large population of non-neuronal cells. Traditional enzymatic and mechanical dissociation methods are ineffective here. Strategies, including 10x Visium spatial transcriptomics and single-nucleus RNA-seq^12–14,44^, have generated pioneering datasets of human DRG neurons. Nevertheless, single-soma transcripts are preferable for cell type clustering and functional interpretation. In the present study, we combined LCM of individual hDRG neuronal soma with Smart-seq2 deep sequencing and employed 10x Xenium spatial transcriptomics. Our dataset overlaps with previous ones to some extent but also exhibits notable differences. Given the high sequencing depth of transcripts from the neuronal soma and the soma size information of dissected hDRG neurons, our dataset is useful for discovering functional molecules expressed at a low level and for accurate cell type clustering. This LCM approach is readily applicable to other human neurons with large soma sizes, such as other neurons of the peripheral nervous system and motor neurons.

We used a 100-customized-probe panel, including 87 top molecular markers for different types of hDRG neurons, to conduct 10x Xenium spatial transcriptomics. The Xenium results unbiasedly clustered the hDRG neurons into functional groups very similar to those obtained by the single-soma RNA-seq method. Our 10x Xenium data revealed spatial clustering within the same population of human DRG neurons, which may reflect neurogenesis wave of the same type in developing DRGs^45^.

Our results suggested that, although broad functional groups of DRG sensory afferents are generally conserved across species, there are noticeable molecular differences. The greatest divergence between mouse and human DRG neurons was observed among C-fiber and A-fiber nociceptors. Mice contain four C-fiber nociceptors (PEP1): two (PEP1.1 and PEP1.2) express TRPV1 but not TRPA1 or PIEZO2, whereas the other two (PEP1.3 and PEP1.4) express all three channels. In humans, this diversity appears to be conflated into two types, hPEP.TRPV1/A1.1 and hPEP.TRPV1/A1.2, which express TRPV1/TRPA1, with or without PIEZO2, respectively.

For A-fiber nociceptors, only two types are identified in mice: the PEP2/CGRP-zeta mechano-heat nociceptive population expressing PIEZO2 and low levels of TRPV1 (refs. ^8,23^), consistent with heat activation only at high temperatures, and the PEP3/CGRP-eta mechano-nociceptive population, conveying fast and sharp pain^7,8^. We identified five types of A-fiber nociceptors in hDRG neurons. Two populations have conserved features: hPEP.CHRNA7 is similar to mouse and macaque PEP2/CGRP-zeta, and hPEP.KIT is similar to mouse and macaque PEP3/CGRP-eta. The other three populations—hPEP.NTRK3, hPEP.PIEZO^h^ and hPEP.0—display greater divergence. The hPEP.PIEZO^h^ cluster is particularly interesting because these neurons express very high levels of *PIEZO2*, unlike any neuron types in the mouse, and likely mediate mechanical sensations in blood vessels and internal organs. Our results suggest that fast pain and interoception are evolutionarily privileged in humans, possibly due to larger body sizes.

There has been a lack of insight into the relationship between functionally and molecularly defined human DRG neurons. We found a strong correspondence between *TRPV1*/*TRPM8* expression and the heat/cold sensitivity of various cutaneous afferents. This suggests that TRPV1 and TRPM8 are the predominant heat and cold sensing molecules in humans. Although the involvement of A-fibers in cold allodynia was demonstrated through nerve block studies^46^, their exact identities remain elusive. Our study suggests that hPEP.KIT neurons, which are A mechano-nociceptors expressing TRPM8, are potential candidates for this function. We also identified that human C-LTMRs were activated by innocuous cooling and warming. The similar mechanical sensitivity of A-LTMRs (field receptors) and C-LTMRs suggests that the cooling response of C-LTMRs is unlikely to be due to cold-induced tissue deformation. It is unclear why C-LTMRs respond to innocuous warmth, whereas C-HTMRs respond to noxious heat, despite both being TRPV1^+^.

With more neurons sequenced, we anticipate discovering even greater heterogeneity among hDRG neurons. In addition, DRGs at different spinal levels innervate different peripheral target tissues^20^. Therefore, sequencing hDRG neurons from different spinal levels and additional donors may uncover differences related to body region, sex, race and age. Moreover, pathological conditions alter the transcriptomic landscape^47,48^. Comparing molecular and cellular changes between donors at the baseline condition (as in this study) and those with chronic itch or pain could increase understanding of the pathological mechanisms and help in identifying molecular targets for effective treatments.

## Methods

### Ethical compliance and declaration

This study complies with all relevant ethical regulations and the revised Declaration of Helsinki. The protocols of collecting skin biopsies from consent human donors were approved by the College of Medicine, University of Florida institutional review board (IRB) committee (IRB201500232 and IRB202300291). The protocol of performing microneurographic recordings with consent human subjects was approved by the Swedish Ethical Review Authority (dnr 2020-04426). The protocol of collecting DRG tissues from consent patients was approved by the University of Pennsylvania IRB committee (IRB834222). The National Disease Research Interchange (NDRI, supported by National Institutes of Health (NIH) grant U42OD11158) has an IRB protocol (IRB704541) for procuring human tissues from postmortem donors, approved by the University of Pennsylvania IRB committee. The Luo laboratory research application was approved by the NDRI Feasibility Committee (RLUW1 01). In addition, as determined by the University of Pennsylvania IRB committee, the Luo laboratory experiments using human DRG samples from de-identified consent postmortem donors were exempted from the human subject requirements.

### Human tissues and subjects

Human DRG tissues used in this study were procured through the NDRI. Tissue recovery was authorized and consented by donor next of kin for research use (unpaid). DRGs were dissected from postmortem donors and immediately frozen in OCT, and the frozen blocks were shipped in dry ice to the Luo laboratory. The Luo laboratory paid the NDRI fees for services. Upon arrival, the tissue blocks were stored at −80 °C until use. Information regarding DRGs, experiments performed, de-identified donors and screening criteria is summarized in Supplementary Tables 1 and 2. In addition, the Luo laboratory received two DRGs from consent patients for the initial pilot experiments through collaboration with the Department of Neurosurgery, University of Pennsylvania. Patients were unpaid for donating their surgically extracted tissues (otherwise discarded) for research.

The human skin biopsies were extracted from three healthy unpaid volunteer donors at the College of Medicine, University of Florida. Information regarding human skin biopsies and de-identified donors is summarized in Supplementary Table 3. These three donors are members of one family and have no noticeable abnormal somatosensation or peripheral neuropathy. All participants were provided written informed consent and signed the document.

In vivo recordings of peripheral sensory afferents of healthy human subjects were performed at Linköping University in Sweden. These subjects were recruited through social media and were compensated for their time at a rate of 200 SEK per hour. All participants provided written informed consent before the start of the experiment.

### LCM of hDRG neurons

The hDRGs imbedded in OCT were cryosectioned (Leica, CM1950 Cryostat) into 20-μm sections and mounted onto Arcturus PEN Membrane Frame Slides (Applied Biosystems, LCM0521). One of every five consecutive sections was collected for LCM to avoid repeated dissection of the soma from the same neuron in different sections. The slides were stored at −80 °C until further use.

On the day of LCM, the slides were transferred to the Skin Biology and Disease Resource-based Center LCM Core on dry ice. Before dissection, the section was briefly stained with RNase-free Arcturu HistoGene staining solution (Applied Biosystems, 12241-05) for better visualization of neuronal soma: 70% cold EtOH for 30 s; HistoGene staining for 10 s; 70% cold EtOH for 30 s; 95% cold EtOH for 30 s; 100% cold EtOH for 30 s; and air dry for 2 min. Then, the slide was put onto an LCM microscope system (Leica, LMD6) for the neuronal soma dissection. The laser was calibrated, and the laser intensity was adjusted to achieve the best dissection efficiency. The dissected individual neuronal soma was collected in the cap of a 200-μl polymerase chain reaction (PCR) tube containing 4 μl of lysis buffer^17^. The sequencing library was generated following the Smart-seq2 workflow^17^. There were three quality control steps before the sequencing. (1) Use housekeeping gene GAPDH (see primer sequences in the supplementary Yu_Material_Reagents file) and neuronal marker PGP9.5 (see primer sequences in the supplementary Yu_Material_Reagents file) to do the RT–PCR after reverse transcription and pre-amplification. The selection standard was the sample with clear and specific GAPDH and PGP9.5 bands. The success rate in this step was 30–60%. (2) Measure the concentration of the sample after the first purification. The selection standard was the sample with concentration greater than 0.2 ng μl^−1^. The success rate in this step was more than 99%. (3) Measure the concentration of the final library. The selection standard was the sample with concentration greater than 1 nM. The success rate in this step was more than 98%. The libraries passing through all quality controls were selected for the final sequencing.

### Sequencing and sequence alignment

The libraries were pooled together (384 samples for one batch) and sequenced on a NovaSeq 6000 platform at the Children’s Hospital of Philadelphia Center for Applied Genomics. Raw sequencing data were demultiplexed with bcl2fastq2 version 2.20 (Illumina) followed by Tn5 transposon adapter sequence trimming with Cutadapt (version 3.5)^49^. The processed reads were then aligned to human genome (GRCh38 GENCODE as the reference genome and GRCh38.104. GTF as the annotation) using STAR version 2.7.9a49 (ref. ^50^). STAR quantMode GeneCounts was used to quantify unique mapped reads to gene counts.

### Analysis of single-soma RNA-seq data of hDRG neurons using Seurat and R

R (version 4.1.2) and Seurat (version 4.0.5) were used for the single-cell RNA-seq analysis. Six objects were created from individual biological replicates. The data were normalized (NormalizeData), after which the 4,500 most variable features were selected (FindVariableFeatures). To mitigate batch effects between replicates, we used Seurat’s integrated analysis approach that transforms datasets into a shared space using pairwise correspondences (or ‘anchors’)^51^. Anchors were first defined using FindIntegrationAnchors (scale = T, normalization.method = LogNormalize, reduction = cca, l2.norm = T, dims = 30, k.anchor = 5, k.filter = 200, k.score = 30, max.features = 200, nn.method = annoy, n.trees = 50, eps = 0), and the data were then integrated (IntegrateData (normalization.method = LogNormalize, features = NULL, features.to.integrate = NULL, dims = 1:30, k.weight = 80, weight.reduction = NULL, sd.weight = 1, sample.tree = NULL, preserve.order = FALSE, eps = 0)) and scaled (ScaleData), followed by principal component analysis (PCA) (RunPCA, npcs = 50). For clustering, the final parameters were as follows: RunUMAP, reduction = pca, dims = 1:25; FindNeighbors, reduction = pca, dims = 1:25; FindClusters, resolution = 3.4. Highly similar clusters without clearly distinguishable markers were merged to produce the final 16 clusters.

### Analysis of single-soma RNA-seq data of hDRG neurons using Conos

For Conos^22^ analysis, single-soma hDRG data were integrated using CCA space $buildGraph (k = 8, k.self = 3, space = CCA, ncomps = 30, n.odgenes = 2000, verbose = TRUE, snn = T, snn.k = 10). For human single-soma and human single-nucleus dataset, co-integration was performed as $buildGraph (k = 8, k.self = 3, space = CCA, n.odgenes = 2000, verbose = TRUE, snn = T, snn.k = 10). For Conos co-clustering, mouse (Sharms) dataset was downsampled to maximum 300 cells per cluster, and co-integration was performed as $buildGraph (k = 8, k.self = 3, space = CCA, n.odgenes = 2000, verbose = TRUE, snn = T, snn.k = 10). Macaque (Kupari, SmartSeq2 dataset) was used for interspecies analysis. For Conos co-clustering, macaque/human graph was built as $buildGraph (k = 4, k.self = 3, space = CCA, ncomps = 30, n.odgenes = 2000, snn = F, snn.k = 10). For all uniform manifold approximation and projection (UMAP) plots in Conos graph embedding was performed as: $embedGraph (method = UMAP, spread = 15, seed = 3). Label propagation ($propagateLabels) was run using the ‘diffusion’ method.

### Methods used to elucidate cross-species cluster relationships

We used four different interspecies analysis approaches. First, we used Conos, which uses graph-based dataset integration and was developed to co-cluster and compare datasets originating from different RNA-seq platforms and species (Fig. 2a,b and Extended Data Fig. 2a,b). Second, we employed a probabilistic neural network analysis, a machine learning approach where the model is trained using one dataset and then applied to other datasets for pattern recognition and probability-based predictions^9^ (Extended Data Fig. 2c,d). Third, we used neural-network-based hierarchical clustering analysis (Extended Data Fig. 2e). In the hierarchical clustering analysis, each query neuron type, either human or macaque, was assigned weights of the sensory type-associated patterns by a neural network, which was trained with gene patterns including both species-specific and shared cross-species features in the different sensory neuron types. The weighted gene patterns were then used for dimensional reduction and nearest neighbor analysis to infer the hierarchical relationship. Finally, we performed TF-GRN analysis across all three species using gene network modules presumably driven by individual TFs (Fig. 2d). These three methods have different strengths and weaknesses. Conos finds shared principal components between integrated datasets, but some species-specific features may be lost and can lead to impaired statistical sufficiency during integration and, furthermore, can be affected by the number of principal components and nearest neighbor distance. Machine learning is based on gene expression and is not supervised by shared latent space (that is, common principal components). Each single reference dataset is used to train the machine learning module and is then tested by the other dataset. Thus, an advantage of this method is that, at the stage of machine learning, datasets are not integrated, and, hence, probability calculations are not affected by principal components or nearest distance. Conversely, its disadvantage is that, because it emphasizes the features of each cell type, the learning accuracy and reliability depend on the robustness of the reference or training dataset. For the third approach, machine-learning-based hierarchical clustering, we extracted the weight of cell-type-specific features to construct the latent space covering all cell types across species, whether shared or not. With this strategy, we tried to obtain sufficient latent space, as compared to Conos, by training and predicting every dataset independently, and, furthermore, parameterization was used to find the most robust hierarchical clustering.

### Cell type probabilistic similarity estimation across cell types and the data integration across species

The assessment of cell type purity, the probabilistic similarity and cell type integration across species were performed using packages in a machine-learning-based single-cell analysis toolkit, scCAMEL, released separately at https://sccamel.readthedocs.io/.

### Probabilistic similarity estimation across cell types

The calculation of cell type probabilistic score is described in the SWAPLINE package^52^. In brief, a vanilla neural network model was built for cell type classification. To train the model, we removed the cell-cycle-related genes and then computed the most variable features. In addition, we ranked the marker genes for each cell type by two heuristics for the cell type specificity of both fold change and enrichment score change. Subsequently, the ranked marker genes and the most variable genes were merged, log transformed and scaled by minimum–maximum normalization for learning models. The frame of the neural network model and the parameters are described in the SWAPLINE package. The learning accuracy of the neural network classifier was inspected against epoch numbers and was estimated by k-fold cross-validation (k = 3). The learning rate and learning epochs were selected according to the maximum point of the learning curve reaching the accuracy plateaus. The probabilistic scores from mouse and macaque species against human reference were visualized in a violin plot.

### Data integration across species

For the integration task, we applied interpretable neural network learning. First, we took one dataset from the dataset pool. We trained a neural network classifier by learning the transcriptional features of each cell type in this dataset and then calculated the probabilistic scores against all cell types. Subsequently, we used all other datasets as query datasets and calculated the probabilistic score of every cell in each query dataset using the trained classifier. Then, we took another dataset from the dataset pool and repeated the training and prediction. We repeated the training and prediction until every dataset had been used as a training reference for the predictions. Here, we considered that the probabilistic score of each cell reflected the weighted gene patterns representing each trained cell type. Thus, we merged the probabilistic scores of all cells from all trained and predicted datasets for the PCA. The most significant principal components were determined by the elbow method and subsequently used as the latent space for further downstream analysis. The tree plot was constructed with the parameter of 11 principal components, 90 nearest neighbors and correlation metric. The trained cell type similarity was calculated with the correlation distance and the average/UPGMA linkage and visualized in the hierarchical heatmap.

In parallel, we normalized the gene expression by interpretable learning. We transformed the gene symbols of each species into the nomenclature in *Homo sapiens*. We estimated the features’ weights in each reference cell type by using the DeepLift algorithm^30^. The gene expression of each cell that had been learned or predicted in one trained reference dataset was inferred by the matrix multiplication between the features’ weights and the cell type probabilistic scores. Then, the final gene × cell expression matrix was calculated by the average of non-empty values across all datasets. Using this normalized expression matrix, we enriched the mostly co-expressed genes using Spearman correlation. These co-expressed genes were used for inferring the TF-associated gene patterns with a modified GENIE3, as described in refs. ^53,54^. The result was visualized as a hierarchical heatmap.

### Methods used to elucidate human DRG neuron cross-dataset cluster relationships

The expression datasets from previously published studies were downloaded and randomly downsampled to match the sample size of our study. The compared two datasets were integrated by FindTransferAnchors (reference, query, dims = 1:30; reference, query, reduction = pca). The assignment possibility of each neuron in the query datasets to the reference cluster was calculated by TransferData (anchorset, refdata, dims = 1:30). The correlation heatmap was generated based on the mean value of prediction in each cluster of the query dataset to each cluster of the reference dataset. The heatmap was plotted by the R package ‘heatmap’.

### 10x Xenium spatial transcriptomics, probes, segmentation and data analysis

Spatial transcriptomics was performed using the Xenium Analyzer (10x Genomics) following the manufacturer’s instructions (https://cdn.10xgenomics.com/image/upload/v1710785024/CG000584_Xenium_Analyzer_UserGuide_RevE.pdf). In brief, 10-μm-thick tissue cryosections were mounted on Xenium slides, and mRNAs were targeted using a custom gene panel consisting of 100 genes, including 87 neuronal marker genes and 13 non-neuronal marker genes (see the list below). Post-Xenium H&E staining was subsequently performed. Cell segmentation was performed manually based on the clustering expression of pan-neuronal marker gene PGP9.5, satellite glia cell marker FABP7 and the corresponding H&E staining. Each transcript was mapped back to the new segmentation for downstream analysis. In total, 87 neuronal marker genes were used for clustering using Seurat (version 4.0.5). The final parameters were as follows: RunUMAP, reduction = pca, dims = 1:10; FindNeighbors, reduction = pca, dims = 1:10; FindClusters, resolution = 2.5. Neurons with bad or unclear neuronal soma morphology from H&E staining were excluded for further analysis. Cell type annotation for each cluster was based on the marker gene expression.

**Table:** 

Gene	Ensembl ID	Gene	Ensembl ID	Gene	Ensembl ID	Gene	Ensembl ID
ADCYAP1	ENSG00000141433	GFRA2	ENSG00000168546	NTRK1	ENSG00000198400	SCG2	ENSG00000171951
ADRA2C	ENSG00000184160	GFRA3	ENSG00000146013	NTRK2	ENSG00000148053	SCG3	ENSG00000104112
AGT	ENSG00000135744	GLDN	ENSG00000186417	NTRK3	ENSG00000140538	SCN10A	ENSG00000185313
ALPL	ENSG00000162551	GPR68	ENSG00000119714	OPRD1	ENSG00000116329	SCN11A	ENSG00000168356
APOE	ENSG00000130203	HRH1	ENSG00000196639	OPRK1	ENSG00000082556	SCN7A	ENSG00000136546
ATF3	ENSG00000162772	HRH2	ENSG00000113749	OPRM1	ENSG00000112038	SCN9A	ENSG00000169432
ATP2B4	ENSG00000058668	HTR3A	ENSG00000166736	OSMR	ENSG00000145623	SCRG1	ENSG00000164106
CACNA2D1	ENSG00000153956	IGFBP7	ENSG00000163453	P2RY1	ENSG00000169860	SEMA6A	ENSG00000092421
CACNA2D2	ENSG00000007402	IL31RA	ENSG00000164509	PCDH7	ENSG00000169851	SERPINA1	ENSG00000197249
CALCA	ENSG00000110680	INA	ENSG00000148798	PCP4	ENSG00000183036	SFRP1	ENSG00000104332
CALCB	ENSG00000175868	KCNA4	ENSG00000182255	PDGFRA	ENSG00000134853	SLC17A6	ENSG00000091664
CASQ2	ENSG00000118729	KCND1	ENSG00000102057	PIEZO1	ENSG00000103335	SLC17A7	ENSG00000104888
CCK	ENSG00000187094	KCNG4	ENSG00000168418	PIEZO2	ENSG00000154864	SLC17A8	ENSG00000179520
CD74	ENSG00000019582	KCNV1	ENSG00000164794	PLCB3	ENSG00000149782	SST	ENSG00000157005
CDH1	ENSG00000039068	KIT	ENSG00000157404	POU4F1	ENSG00000152192	STUM	ENSG00000203685
CHRNA3	ENSG00000080644	MGP	ENSG00000111341	PRDM12	ENSG00000130711	SYP	ENSG00000102003
CHRNA7	ENSG00000175344	MPZ	ENSG00000158887	PTGDR	ENSG00000168229	TAC1	ENSG00000006128
CPNE4	ENSG00000196353	MRGPRD	ENSG00000172938	PTGER3	ENSG00000050628	TINAGL1	ENSG00000142910
CST3	ENSG00000101439	MRGPRX1	ENSG00000170255	PTGIR	ENSG00000160013	TMEM273	ENSG00000204161
DCN	ENSG00000011465	MRGPRX3	ENSG00000179826	PTPRC	ENSG00000081237	TRPA1	ENSG00000104321
DOC2A	ENSG00000149927	MRGPRX4	ENSG00000179817	PVALB	ENSG00000100362	TRPM8	ENSG00000144481
EDNRA	ENSG00000151617	NEFH	ENSG00000100285	REEP5	ENSG00000129625	TRPV1	ENSG00000196689
EHD3	ENSG00000013016	NEFL	ENSG00000277586	RET	ENSG00000165731	UCHL1	ENSG00000154277
ENTPD3	ENSG00000168032	NMB	ENSG00000197696	S100A16	ENSG00000188643	UCN	ENSG00000163794
FABP7	ENSG00000164434	NPPB	ENSG00000120937	S100A4	ENSG00000196154	WLS	ENSG00000116729

### Statistical analysis of hDRG neuron cell clustering probability

The area $A$ of the hDRG section containing neurons was calculated using ImageJ software. For each neuron cell type with $N$ cells, the unit area for each cell is $a=A/N$. The centroid for each cell was identified based on its cell boundary. We were interested in examining if cells from the same cell type tended to be spatially near each other. To examine this, for each cell, we define its neighborhood by drawing a circle centered around the cell centroid with radius $r$. In our analysis, we set $r=250{um}$. If there is no spatial enrichment of cells from the same cell type in the neighborhood, we expect to get ${n}_{\exp }=\pi \times {r}^{2}/a$ cells from the same cell type within the circle. The number of observed cells of the same cell type in the neighborhood, denoted by ${n}_{{obs}}$, was counted by including cells whose Euclidian distance with the center cell was less than $r$. To test if there was an enrichment of cells of the same cell type in the given cell’s neighborhood, we calculated the chi-squared statistic as ${({n}_{{obs}}-{n}_{\exp })}^{2}/{n}_{\exp }$, and the corresponding *P* value was calculated using function ${\rm{pchisq}}()$ in the R ‘stats’ package with degree of freedom set to 1. Repeating this analysis for every cell in a given cell type, we obtained the *P* value distribution for each cell type. The *P* value distribution was visualized using ${\rm{geom\_histogram}}()$ function in the R ‘ggplot2’ package.

To determine if the *P* value distribution was enriched toward 0, we focused on cell types with *n* > 50. We first generated a group of random numbers of length $N$ from uniform distribution between 0 and 1, and then we performed two-sample one-sided *t*-tests to compare the observed *P* values with these randomly generated numbers in −log_10_ scale using the t.test() function in the R ‘stats’ package. We repeated this one-sided *t*-test 1,000 times and calculated the mean *P* value for each cell type. Our results indicated that the mean *P* values were less than 0.05 for all 12 considered cell types after multiple testing correction, indicating that cells from the same cell type were spatially near each other.

### RNAscope multiplex FISH, confocal microscopy imaging and quantification

OCT-embedded freshly dissected human lumbar or thoracic DRG tissues were cryosectioned at 20-µm thickness and mounted on glass slides. The slides were stored at −80 °C to preserve RNA integrity. RNAscope Fluorescent Multiplex Reagent Kit and RNAscope probes for the targeted genes (Advanced Cell Diagnostics) were used for multiplex FISH. RNAscope in situ hybridization was performed in accordance with the manufacturer’s instructions. In brief, fresh frozen hDRG sections were fixed, dehydrated and treated with protease. The sections were then hybridized with the respective target probe for 2 h at 40 °C, followed by 2–3 rounds of signal amplification. The sections were then mounted under coverslips, sealed with nail polish and stored in the dark at 4 °C until imaged. A Leica SP5 confocal microscope was used to capture images, and ImageJ was used for image analysis. The criteria for defining a cell as positive for a particular channel/gene were as follows. The real RNAscope signals should be composed of small fluorescent dots (>10) in a cell area, as opposed to the surrounding areas or in other negative cells, and they should not completely overlap with signals from all other channels. In some DRG neurons, accumulation of lipofuscin in part of cells caused strong autofluorescence visible in all channels. These signals were considered as non-specific background and were excluded for analysis. The percentage of each cluster over all DRG neurons could be somewhat overestimated due to the following two reasons: (1) some marker genes or marker gene combinations were not specific and may also label a small subset of other cell types; and (2) an underestimation in quantification of total neuronal numbers because some cells had neither multiple FISH signals nor DAPI nucleus staining signals.

### Human skin biopsy extraction, processing and immunostaining

In brief, 1 cc of lidocaine was injected subdermally at each biopsy location (Supplementary Table 3). A total of six 3-mm dermal skin punch biopsies were performed on each of the three subjects. Excised skin was immediately placed in 1.5-ml Eppendorf tubes containing 4 °C 4% paraformaldehyde (PFA) solution that was freshly prepared on the same day of the skin biopsy procedure. Biopsy tissue was fixed in 4% PFA (dissolved in PBS) for exactly 4 h at 4 °C, followed by 2 × 30-min washes in PBS solution and then cryoprotected using 1× PBS and 30% sucrose at 4 °C. These tissues in cold 1×PBS, 30% sucrose were overnight shipped to the laboratory of Integrated Tissue Dynamics.

The skin biopsies were mounted in OCT and cryosectioned into 14-μm sections. Adjacent sections were collected by continuous slides. Immunofluorescence was performed using combinations of mouse monoclonal anti-human PGP9.5 (Protein Gene Product, Cedarlane, 31A3 (source UltraClone), 1:200), sheep polyclonal anti-human CGRP (Abcam, ab195387, lot no. GR3434340-7, 1:500), mouse monoclonal anti-human NEFH (Sigma-Aldrich, n0142, 1:400), rabbit anti-human SST (ImmunoStar, 20067) and anti-human C-KIT (Abcam, ab32363, lot no. GR294191-14, 1:200). Slides were pre-incubated in 1% BSA and 0.3% Triton X-100 in PBS (PBS-TB) for 30 min and then incubated with primary antibodies diluted in PBS-TB overnight in a humid atmosphere at 4 °C. Slides were then rinsed in excess PBS for 30 min and incubated for 2 h at room temperature with the appropriate secondary antibodies diluted in PBS-TB (Jackson ImmunoResearch, Cy3 donkey anti-rabbit IgG, 711-165-152, 1:500; Life Technologies, Alexa Fluor 488 donkey anti-mouse IgG, A21202, 1:250 and Alexa Fluor 488 donkey anti-sheep IgG, A11015, 1:250). After secondary antibody incubation, the sections were rinsed for 30 min in PBS and coverslipped under 90% glycerol in PBS. Images were collected using a ×20 objective on an Olympus BX51-WI microscope equipped with conventional fluorescence filters (Cy3:528–553 nm excitation, 590–650 nm emission; Cy2/Alexa Fluor 488: 460–500 nm excitation, 510–560 nm emission), a Hamamatsu ER, a DVC high-speed camera, a linear focus encoder and a three-axis motorized stage system interfaced with Neurolucida software (MBF Bioscience).

### In vivo electrophysiological recording of human peripheral sensory fibers

Single-unit axonal recordings (microneurography) were performed from the right posterior antebrachial cutaneous, radial or superficial peroneal nerve of 62 healthy participants (29 males and 33 females, 19–41 years of age). Participants were comfortably seated in an adjustable chair with legs and arms stretched out (and hand pronated), supported by vacuum pillows and covered in a blanket if they reported feeling cold.

Neural activity was sampled at 20 kHz and recorded using the ADInstruments data acquisition system (LabChart software version 8.1.24 and PowerLab 16/35 hardware, PL3516/P) and then exported to Spike2 (version 10.13, Cambridge Electronic Design). Single action potentials were identified semiautomatically, with visual verification on an expanded timescale. Threshold crossing was used to distinguish action potentials from noise with a signal-to-noise ratio of at least 2:1, and spike morphology was confirmed by template matching. Recordings were discarded if multiple units were present (for example, non-physiological spike intervals/firing rates) or if spike amplitudes were not distinct from the noise, preventing secure action potential identification. The neural response was always spatially locked—that is, evoked (or modulated) only when the specific area of skin (the receptive field) was stimulated. Furthermore, repeat trials for each stimulus were conducted to ensure reproducibility.

Under real-time ultrasound guidance (LOGIQ P9, GE Healthcare), the target nerve was impaled with an insulated tungsten recording electrode (FHC, Inc.). Adjacent to that, an uninsulated reference electrode was inserted just under the skin. A high-impedance pre-amplifier (MLT185 Headstage, ADInstruments) was attached to the skin near the recording electrode and used together with a low-noise, high-gain amplifier (FE185 Neuro Amp EX, ADInstruments). Once the electrode tip was intrafascicular, single LTMRs were searched for by soft brush stroking, and single HTMRs were searched for by coarse brush stroking, pinching and hair tugging in the fascicular innervation zone while making minute electrode adjustments.

All recorded afferents were mechanically responsive and divided into subtypes based on established criteria^36,38,55^. Mechanical threshold and receptive field size were determined using Semmes–Weinstein monofilaments (nylon fiber; Aesthesio, BIOSEB). Mechanical threshold was defined as the weakest monofilament to which the unit responded in at least 50% of trials. The conduction velocity of the recorded afferent was estimated from latency responses to surface electrical stimulation (FE180 Stimulus Isolator, ADInstruments) or rapid mechanical tapping using an electronic filament (Physiology Section, Department of Integrative Medical Biology, Umeå University) at the receptive field. Electrically and mechanically evoked spikes were compared on an expanded timescale to confirm that they originated from the same unit. Thermal responsiveness was tested by placing a Peltier probe (T09 and T10, QST.Lab) onto the receptive field. After recording baseline activity for at least 30 s (with the thermode in contact with the receptive field) at a neutral temperature of 30 °C, a series of cooling (down to 0 °C at 4 °C s^−1^) and warming (up to 50 °C at 4 °C s^−1^) stimuli were delivered at 30-s intervals. If needed, the thermode was mounted on a stand for better stability.

To test *TRPV1* expression, capsaicin (Capsina 0.075%, Bioglan AB) was topically applied to the receptive field. After 1 min, the skin was wiped clean, and the emergence of any spontaneous spiking activity from the recorded afferent was monitored. *TRPM8* expression was tested by placing an ethanol-soaked gauze pad (90% ethanol as control) onto the receptive field, followed by menthol solution (400 mg of 40% L-menthol dissolved in 90% ethanol, Sigma-Aldrich^56^). The gauze pad was covered with an adhesive film to prevent evaporation of ethanol. After 5 min, the skin was wiped clean, and the emergence of any spontaneous spiking activity from the recorded afferent was monitored.

### Statistics and reproducibility

All data shown in column and line graphs represent mean ± s.e.m., unless otherwise mentioned. Significance levels are indicated as *P < 0.05; **P < 0.01; ***P < 0.001; and ****P < 0.0001. Sample sizes, statistical methods and repeatability are mentioned in respective figure legends.

For human physiological recordings, no statistical methods were used to predetermine sample size, but our sample sizes are similar to those reported in previous publications^38,57,58^. No data were excluded from the analyses. The experiments were not randomized. Data collection and analysis were not performed blinded to the conditions of the experiments. Data distribution was tested to inform the choice between parametric or non-parametric tests, with the specific test mentioned alongside the statistical results in the figure legends.

### Figure generation software

Figures were generated in PowerPoint (Microsoft Office) and GraphPad Prism (versions 8 and 9, GraphPad Software). Some cartoons were made partially in BioRender (BioRender, 2022, RRID: SCR_018361).

### Reporting summary

Further information on research design is available in the Nature Portfolio Reporting Summary linked to this article.

## Online content

Any methods, additional references, Nature Portfolio reporting summaries, source data, extended data, supplementary information, acknowledgements, peer review information; details of author contributions and competing interests; and statements of data and code availability are available at 10.1038/s41593-024-01794-1.

## Supplementary information

Supplementary Information Supplementary Tables 1–3.

Reporting Summary

Supplementary Data 1 List of key materials and reagents.

## Source data

Source Data main figures Statistical source data for Figs. 1–8.

Source Data extended data figures Statistical source data for Extended Data Figs. 1–10.

## Data Availability

The raw and processed datasets for the single-soma sequencing of hDRG neurons were deposited in the Gene Expression Omnibus (GEO) (GSE249746), and the raw and processed datasets for the 10x Xenium transcriptomics of hDRG neurons were deposited in the GEO (GSE273557) and Dryad (10.5061/dryad.gf1vhhmxq). A publicly accessible website is available using Shiny app interphase containing the processed data for browsing and searching gene expression in the different human neuron types (https://ernforsluolabs.shinyapps.io/HumanDRG/). Macaque (Kupari) data are available at https://www.ncbi.nlm.nih.gov/geo/query/acc.cgi?acc=GSE165569 Mouse (Zeisel) DRG data are available at http://loom.linnarssonlab.org/clone/Mousebrain.org.level6/L6_Peripheral_sensory_neurons.loom. Mouse (Sharma) DRG data are available at https://www.ncbi.nlm.nih.gov/geo/query/acc.cgi?acc=GSE139088 Human (Tavares-Ferreira) DRG data are available at https://www.ncbi.nlm.nih.gov/projects/gap/cgi-bin/study.cgi?study_id=phs001158.v2.p1 Human (Nguyen) DRG data are available at https://www.ncbi.nlm.nih.gov/geo/query/acc.cgi?acc=GSE168243 Human (Jung) DRG data are available at https://www.ncbi.nlm.nih.gov/geo/query/acc.cgi?acc=GSE201654 GRCh38 GENCODE database (genome sequence alignment reference) is available at http://hgdownload.cse.ucsc.edu/goldenPath/hg38/bigZips/hg38.fa.gz GRCh38.104. GTF database (human genome annotation reference) is available at https://ftp.ensembl.org/pub/release-104/gtf/homo_sapiens/. Source data are provided with this paper.

## Code availability

Custom code has been deposited to GitHub (https://github.com/taimeimiaole/NN_hDRG-neuron-sequencing). Previously published codes and software used for this study are cited in the Methods.
