# Author Correction: Leveraging deep single-soma RNA sequencing to explore the neural basis of human somatosensation

**DOI:** 10.1038/s41593-026-02249-5

**Published:** 2026-03-06

**Authors:** Huasheng Yu, Saad S. Nagi, Dmitry Usoskin, Yizhou Hu, Jussi Kupari, Otmane Bouchatta, Hanying Yan, Suna Li Cranfill, Mayank Gautam, Yijing Su, You Lu, James Wymer, Max Glanz, Phillip Albrecht, Hongjun Song, Guo-Li Ming, Stephen Prouty, John Seykora, Hao Wu, Minghong Ma, Andrew Marshall, Frank L. Rice, Mingyao Li, Håkan Olausson, Patrik Ernfors, Wenqin Luo

**Affiliations:** 1 Department of Neuroscience, Perelman School of Medicine, University of Pennsylvania, Philadelphia, PA USA; 2 Department of Biomedical and Clinical Sciences, Linköping University, Linköping, Sweden; 3 Department of Medical Biochemistry and Biophysics, Division of Molecular Neurobiology, Karolinska Institute, Stockholm, Sweden; 4 Department of Biostatistics in Biostatistics and Epidemiology, Perelman School of Medicine, University of Pennsylvania, Philadelphia, PA USA; 5 Department of Biochemistry and Molecular Biology, College of Medicine, University of Florida, Gainesville, FL USA; 6 Neuroscience & Pain Research Group, Integrated Tissue Dynamics, LLC, Rensselaer, NY USA; 7 Department of Dermatology, Perelman School of Medicine, University of Pennsylvania, Philadelphia, PA USA; 8 Department of Genetics, Perelman School of Medicine, University of Pennsylvania, Philadelphia, PA USA; 9 Pain Research Institute, Institute of Life Course and Medical Sciences, University of Liverpool, Liverpool, UK

**Keywords:** Sensory processing, Somatic system

Correction to: *Nature Neuroscience* 10.1038/s41593-024-01794-1, published online 4 November 2024.

In the version of this article initially published, several figure elements were incorrect. In Fig. 4c, the labels “hPEP.PIEZo^h^”, “hAb.LTMR”, and “hAd.LTMR” should have read “hPEP.PIEZO^h^”, “hAβ.LTMR”, and “hAδ.LTMR”, respectively. In Fig. 4d, the positions of the x-axis labels “CHRNA7” and “KIT” were swapped. In Fig. 4e, the panels for “hPEP.NTRK3” and “hAβ.LTMR” inadvertently showed the same image; the “hAβ.LTMR” panel has been replaced with the correct image. In Fig. 8i, the label “C-HTMR Heat^+^” should be “C-HTMR Cool^+^.” Also, in Fig. 8i, the axis tick label “20” was a typographical concatenation and should appear as two separate ticks, “2” and “0.” In Fig. 8i–j, the label “noxious range” has been removed; the grey shading is intended only to indicate the sustained phase (already denoted by “0 °C sustained” and “50 °C sustained”). These errors have been corrected in the HTML and PDF versions of the article.

