# Supplementary material for: Leveraging deep single-soma RNA sequencing to explore the neural basis of human somatosensation
Source: Nat Neurosci. 2024 Nov 4;27(12):2326–40. doi: 10.1038/s41593-024-01794-1 (PMC11614738; doi:10.1038/s41593-024-01794-1)
Supplement: Supplementary file 1 — Supplementary Tables 1–3. [file 41593_2024_1794_MOESM1_ESM.pdf]

nature neuroscience

Supplementary information https://doi.org/10.1038/s41593-024-01794-1

Leveraging deep single-soma RNA sequencing to explore the neural basis of human somatosensation

In the format provided by the authors and unedited

Supplementary Table 1. Summary of donor information for DRG samples

Donor	Age	Sex	Race	Tissue ID	DRG level	Ventilator	Recovery interval	Conducted experiments
1	61	female	White	N2-RL5	Lumbar 5	Yes	1.3 hours	LCM
				N2-RT12	Thoracic 12			LCM
2	56	female	White	N3-RL2	Lumbar 2	Yes	6.9 hours	LCM + Xenium + RNAscope
				N3-RT11	Thoracic 11			LCM
3	23	male	White	N4-RL3	Lumbar 3	Yes	1.3 hours	LCM + Xenium
				N4-RT12	Thoracic 12			LCM + Xenium + RNAscope

Supplementary Table 2. Donor screening criterion for human DRG samples

Age Range	18-65
Race	Any
Sex	No Preference
Required Disease(s)	Normal
Required Medication(s)	None
Required Surgeries(s)	None
Infectious Disease Testing Required	Yes
Sepsis	No
Increased Risk	Some Acceptable If Infectious Disease Negative
Acceptable Warm Ischemic Time (WIT)	Less than 60
Acceptable Downtime	Less than 60
Vent Time	Always Acceptable
Cancer	Never Acceptable
Chemotherapy	Never Acceptable
Radiation	Never Acceptable
Unacceptable Diseases	Diabetes - Type I (T1D, T1DM)<br>Diabetes - Type II (T2D, T2DM)<br>Diabetes
Acceptable Diseases	None
Unacceptable Surgeries	None
Acceptable Surgeries	None
Unacceptable Medications	None
Acceptable Medications	None
Tobacco Use	Always Acceptable
Alcohol Use	Some Acceptable<br>Quantity: 2 Drinks per Day<br>Day Duration: No Restrictions<br>Use Category: Moderate Drinking<br>Time Since last use: No Restrictions
Illicit Drug Use	Never Acceptable

Supplementary Table 3. Summary of donor information for human skin samples

Donor	Age	Sex	Race	Tissue ID	DRG level	Date of sampling
1	60	female	White	868-01	Left Ankle	03/27/2023
				868-02	Left Thigh	
				868-03	Left Hand	
				868-04	Right Ankle	
				868-05	Right Thigh	
				868-06	Right Hand	
2	26	female	White	892-01	Left Hand	05/15/2023
				892-02	Left Thigh	
				892-03	Left Ankle	
				892-04	Right Hand	
				892-05	Right Thigh	
				892-06	Right Ankle	
3	24	male	White	793-01	Left Hand	07/14/2023
				793-02	Right Hand	
				793-03	Right Thigh	
				793-04	Left Thigh	
				793-05	Left Ankle 1	
				793-06	Left Ankle 2	
