# Supplementary material for: Leveraging deep single-soma RNA sequencing to explore the neural basis of human somatosensation
Source: Nat Neurosci. 2024 Nov 4;27(12):2326–40. doi: 10.1038/s41593-024-01794-1 (PMC11614738; doi:10.1038/s41593-024-01794-1)
Supplement: Supplementary file 2 — Reporting Summary [file 41593_2024_1794_MOESM2_ESM.pdf]

nature portfolio

Corresponding author(s): Wenqin Luo

Last updated by author(s): Aug 14, 2024

Reporting Summary

Nature Portfolio wishes to improve the reproducibility of the work that we publish. This form provides structure for consistency and transparency in reporting. For further information on Nature Portfolio policies, see our Editorial Policies and the Editorial Policy Checklist.

Statistics

For all statistical analyses, confirm that the following items are present in the figure legend, table legend, main text, or Methods section.

n/a	Confirmed	
☐	☒	The exact sample size (*n*) for each experimental group/condition, given as a discrete number and unit of measurement
☐	☒	A statement on whether measurements were taken from distinct samples or whether the same sample was measured repeatedly
☐	☒	The statistical test(s) used AND whether they are one- or two-sided<br>*Only common tests should be described solely by name; describe more complex techniques in the Methods section.*
☐	☒	A description of all covariates tested
☐	☒	A description of any assumptions or corrections, such as tests of normality and adjustment for multiple comparisons
☐	☒	A full description of the statistical parameters including central tendency (e.g. means) or other basic estimates (e.g. regression coefficient) AND variation (e.g. standard deviation) or associated estimates of uncertainty (e.g. confidence intervals)
☐	☒	For null hypothesis testing, the test statistic (e.g. *F*, *t*, *r*) with confidence intervals, effect sizes, degrees of freedom and *P* value noted<br>*Give P values as exact values whenever suitable.*
☒	☐	For Bayesian analysis, information on the choice of priors and Markov chain Monte Carlo settings
☒	☐	For hierarchical and complex designs, identification of the appropriate level for tests and full reporting of outcomes
☒	☐	Estimates of effect sizes (e.g. Cohen's *d*, Pearson's *r*), indicating how they were calculated

Our web collection on statistics for biologists contains articles on many of the points above.

Software and code

Policy information about availability of computer code

Data collection	The LCM images data were collected using Leica LMD6 microscope system<br>The sequencing data were collected using NovaSeq 6000 platform.<br>The Xenium data were collected using Xenium Analyzer and Aperio Scanner microscope system.<br>RNAScope date were collected using Leica SP5 confocal microscope and LAS X Leica Microsystems.<br>Immunofluorescence data were collected in LOlympus BX51-WI microscope.<br>Human in vivo recording data were collected using the ADInstruments data acquisition system.<br>Other data were collected in MS office (Version 2405).
Data analysis	Single soma RNA-seq alignment in STAR (v.2.7.9a49).<br>Tn5 transposon adapter sequences trimming with Cutadapt (V3.5)<br>Single-soma RNA-seq data analysis in R (version 4.1.2), Seurat (version 4.0.5), Conos, scCAMEL and SWAPLINE packages.<br>Statistical tests and data analysis in Electrophysiology study were performed by Clampfit and Prism 8 (Commercial softwares).<br>Immunofluorescence, RNAScope and Xenium imaging data were also analyzed by Fiji/ImageJ (v2.3.0;NIH).<br>In vivo electrophysiological recording data using Spike2 (v10.13)<br>hDRG neuron cell clustering analysis using R (version 4.1.2).<br>Rest of the data were analyzed using Graph Pad Prism 9.<br>Custom codes for data analysis were uploaded to Github: https://github.com/taimeimiaole/NN_hDRG-neuron-sequencing

For manuscripts utilizing custom algorithms or software that are central to the research but not yet described in published literature, software must be made available to editors and reviewers. We strongly encourage code deposition in a community repository (e.g. GitHub). See the Nature Portfolio guidelines for submitting code & software for further information.

Data

Policy information about availability of data

All manuscripts must include a data availability statement. This statement should provide the following information, where applicable:

- Accession codes, unique identifiers, or web links for publicly available datasets
- A description of any restrictions on data availability
- For clinical datasets or third party data, please ensure that the statement adheres to our policy

Data availability
The raw and processed datasets for the single-soma sequencing of hDRG neurons were deposit-ed in GEO (GSE249746), and the raw and processed datasets for the 10x Xenium transcriptom-ics of hDRG neurons were deposited in GEO (GSE273557) and Dryad (DOI: 10.5061/dryad.gf1vhhmxq). A publicly accessible website using Shinyapp interphase containing the processed data for browsing and searching gene expression in the different human neuron types (https://ernforsluolabs.shinyapps.io/HumanDRG/) will be available once the manuscript is ac-cepted for publication.
Macaque (Kupari) data is available at
https://www.ncbi.nlm.nih.gov/geo/query/acc.cgi?acc=GSE165569
Mouse (Zeisel) DRG data is available at
http://loom.linnarssonlab.org/clone/Mousebrain.org.level6/L6_Peripheral_sensory_neurons.loom. Mouse (Sharma) DRG data is available at
https://www.ncbi.nlm.nih.gov/geo/query/acc.cgi?acc=GSE139088
Human (Tavares-Ferreira) DRG data is available at
https://www.ncbi.nlm.nih.gov/projects/gap/cgi-bin/study.cgi?study_id=phs001158.v2.p1
Human (Nguyen) DRG data is available at
https://www.ncbi.nlm.nih.gov/geo/query/acc.cgi?acc=GSE168243
Human (Jung) DRG data is available at
https://www.ncbi.nlm.nih.gov/geo/query/acc.cgi?acc=GSE201654
GRCh38 GENCODE database (genome sequence alignment reference) is available at
http://hgdownload.cse.ucsc.edu/goldenPath/hg38/bigZips/hg38.fa.gz
GRCh38.104. GTF database (human genome annotation reference) is available at
https://ftp.ensembl.org/pub/release-104/gtf/homo_sapiens/

Research involving human participants, their data, or biological material

Policy information about studies with human participants or human data. See also policy information about sex, gender (identity/presentation), and sexual orientation and race, ethnicity and racism.

Reporting on sex and gender	The terms "male" and "female" used in this manuscript mean sex or biological attribute. We got de-identified postmortem donor information from NDRI.
Reporting on race, ethnicity, or other socially relevant groupings	Yes for postmortem donors. No for human subjects participating in mng recording. Sweden law prohibits collecting race-related information.
Population characteristics	62 healthy consent participants include 29 males and 33 females, ages are from19 to 41 years for mng recording. Three consent adult (24, 26, and 60, two females and one male) donors for the human skin biopsy.
Recruitment	The human skin biopsies were extracted from three healthy unpaid volunteer donors at the College of Medicine, University of Florida. These three donors are members of one family and have no noticeable abnormal somatosensation or peripheral neuropathy. All participants were provided written informed consent and signed the document.<br>In vivo recordings of peripheral sensory afferents of healthy human subjects were performed at Linköping University, Sweden. These subjects were recruited through social media and were compensated for their time at a rate of 200 SEK per hour. All participants provided written informed consent before the start of the experiment. No potential self-selection bias or other bias were noticed during the recruitment
Ethics oversight	The protocols of collecting skin biopsies from consent human donors were approved by the College of Medicine, University of Florida Institutional Review Board (IRB) committee (IRB201500232 and IRB202300291). The protocol of performing microneurographic recordings with consent human subjects was approved by the Swedish Ethical Review Authority (dnr 2020-04426). The protocol of collecting Dorsal Root Ganglion (DRG) tissues from consent patients was approved by the University of Pennsylvania IRB committee (IRB834222). In addition, as determined by the University of Pennsylvania IRB committee, the study using human DRG samples from de-identified consent post-mortem donors was exempted from the human subject requirements.

Note that full information on the approval of the study protocol must also be provided in the manuscript.

Field-specific reporting

Please select the one below that is the best fit for your research. If you are not sure, read the appropriate sections before making your selection.

☒ Life sciences ☐ Behavioural & social sciences ☐ Ecological, evolutionary & environmental sciences

For a reference copy of the document with all sections, see nature.com/documents/nr-reporting-summary-flat.pdf

Life sciences study design

All studies must disclose on these points even when the disclosure is negative.

Sample size	For human skin immunostaining,skin biopsies were extracted from three healthy donor for reproducibility. For human physiological recordings, no statistical methods were used to predetermine sample sizes but our sample sizes are similar to those reported in previous publications (PMID: 7776258, PMID: 19363489, 10.1109/TAFFC.2024.3435060).
Data exclusions	We sequenced 1136 neurons total and excluded 70 neurons for the final analysis due to obvious transcriptome contamination from glia cells.
Replication	All experiments were repeated at least once, with detailed information provided in the figure legends or methods section of the manuscript. We confirmed the reproducibility of the experimental findings, and all replication attempts were successful.
Randomization	We randomly dissected human DRG neurons. For human physiological recording, the participants were not randomized as this is not relevant to your study. We randomly selected sensory fibers in human subjects for physiological recordings.
Blinding	We did not compare different groups. Blinding is not relevant to the study.

Reporting for specific materials, systems and methods

We require information from authors about some types of materials, experimental systems and methods used in many studies. Here, indicate whether each material, system or method listed is relevant to your study. If you are not sure if a list item applies to your research, read the appropriate section before selecting a response.

Materials & experimental systems

n/a	Involved in the study	
☐	☒	Antibodies
☒	☐	Eukaryotic cell lines
☒	☐	Palaeontology and archaeology
☒	☐	Animals and other organisms
☒	☐	Clinical data
☒	☐	Dual use research of concern
☒	☐	Plants

Methods

n/a	Involved in the study	
☒	☐	ChIP-seq
☒	☐	Flow cytometry
☒	☐	MRI-based neuroimaging

Antibodies

Antibodies used	Primary antibodies:<br>mouse monoclonal anti-human PGP9.5 (CedarLane #31A3)<br>sheep polyclonal anti-human CGRP (Abcam #ab195387, lot#GR3434340-7)<br>mouse monoclonal anti-human NEFH (Sigma #n0142)<br>rabbit anti-human SST (ImmunoStar 20067)<br>anti-human KIT (Abcam #ab32363)<br><br>Secondary antibodies:<br>Jackson Immuno Research, West Grove PA 19390, Cy3 donkey anti-rabbit IgG, #711-165-152<br>Life Technologies, Carlsbad CA 92008, Alexa488 donkey anti-mouse IgG, #A21202<br>Alexa488 donkey anti-sheep IgG, #A11015
Validation	Validations from the manufacturer's websites or the previous published work. Mouse monoclonal anti-human PGP9.5 was validated in the website (https://www.abnova.com/en-global/product/detail/MAB11258); Sheep polyclonal anti-human CGRP was validated by previous publication (https://www.abcam.com/en-us/products/primary-antibodies/cgrp-antibody-ab195387); Mouse monoclonal anti-human NEFH was validated by previous publication (https://www.sigmaaldrich.com/US/en/product/sigma/n0142); Rabbit anti-human SST as validated by previous publication (https://www.citeab.com/antibodies/677123-20067-somatostatin-antibody); Anti-human KIT was validated in the website and by previous publication (https://www.abcam.com/en-us/products/primary-antibodies/c-kit-antibody-yr145-ab32363)

Plants

Seed stocks	N.A.
Novel plant genotypes	N.A.
Authentication	N.A.
